# Locoregional Therapies for Hepatocellular Carcinoma: A Systematic Review and Meta-Analysis

**DOI:** 10.1001/jamanetworkopen.2024.47995

**Published:** 2024-11-27

**Authors:** Krishnan R. Patel, Hari Menon, Roshal R. Patel, Erich P. Huang, Vivek Verma, Freddy E. Escorcia

**Affiliations:** 1 Radiation Oncology Branch, National Cancer Institute, National Institutes of Health, Bethesda, Maryland; 2 Department of Human Oncology, University of Wisconsin, Madison; 3 Department of Radiation Oncology, Memorial Sloan-Kettering Cancer Center, New York, New York; 4 Biometric Research Program, National Cancer Institute, Bethesda, Maryland; 5 Department of Radiation Oncology, The University of Texas MD Anderson Cancer Center, Houston

## Abstract

**Importance:**

Several locoregional therapies (LRTs) for nonmetastatic hepatocellular carcinoma (HCC) are available; however, a global comparison of the relative efficacy of each is needed.

**Objective:**

To conduct a systematic review and direct, pairwise meta-analytic comparison of all identified randomized clinical trials evaluating the treatment of nonmetastatic HCC.

**Data Sources:**

A comprehensive search of PubMed and the proceedings of the American Society of Clinical Oncology and American Society for Radiation Oncology annual meetings from January 1, 2010, to November 1, 2023, was performed.

**Study Selection:**

Randomized clinical trials using a form of LRT (surgery with or without adjuvant therapy, radiofrequency ablation [RFA], microwave ablation [MWA], radiotherapy [RT], hepatic arterial infusion chemotherapy [HAIC], transarterial bland embolization [TAE], transarterial chemoembolization [TACE], or transarterial radioembolization [TARE]).

**Data Extraction and Synthesis:**

Study eligibility and data extraction were each reviewed by 2 authors independently. Random-effects meta-analyses were used to compare treatment categories.

**Main Outcomes and Measures:**

Progression-free survival (PFS) was the primary outcome; overall survival (OS) was the secondary outcome.

**Results:**

Forty randomized clinical trials reporting on comparative outcomes of 11 576 total patients with localized HCC treated with LRT were included. The median follow-up was 30.0 (IQR, 18.5-40.8) months. Direct pooled comparisons between treatment classes suggested improved outcomes for surgery combined with adjuvant therapy over surgery alone (PFS: hazard ratio [HR], 0.62 [95% CI, 0.51-0.75]; *P* < .001; OS: HR, 0.61 [95% CI, 0.48-0.78]; *P* < .001), surgery over RFA (PFS: HR, 0.74 [95% CI, 0.63-0.87]; *P* < .001; OS: HR, 0.71 [95% CI, 0.54-0.95]; *P* = .02), RT over TACE (PFS: HR, 0.35 [95% CI, 0.21-0.60]; *P* < .001; OS: HR, 0.35 [95% CI, 0.13-0.97]; *P* = .04), and HAIC over TACE (PFS: HR, 0.57 [95% CI, 0.45-0.72]; *P* < .001; OS: HR, 0.58 [95% CI, 0.45-0.75]; *P* < .001). No substantial heterogeneity was noted for any pairwise comparison with the exception of RT-based regimens compared with tyrosine kinase inhibitor therapy.

**Conclusions and Relevance:**

The findings of this systematic review and direct, pairwise meta-analysis suggest that all LRTs are not equivalent for the treatment of localized HCC. The efficacy of LRTs appears hierarchical, with surgery-based management outcomes associated with the best treatment outcomes and embolization-based treatment options associated with the worst treatment outcomes.

## Key Points

**Question:**

Which locoregional therapy (LRT) or combination of LRT and systemic therapy is preferred for patients with nonmetastatic hepatocellular carcinoma?

**Findings:**

In this meta-analysis of 40 randomized clinical trials (11 576 total patients), evidence for differences in the efficacy between various forms of LRT was observed on the end points of progression-free and overall survival, and a hierarchical structure emerged. Surgical-based LRTs were associated with the greatest benefit, and embolization-based LRTs were associated with poorer outcomes on these end points.

**Meaning:**

These findings suggest that LRT remains an important tool in treating hepatocellular carcinoma and, for patients eligible for LRTs, some forms of LRT may be favored over others.

## Teaser

This systematic review and meta-analysis compares findings from randomized clinical trials on the use of locoregional therapies as monotherapy or combination therapy in the treatment of nonmetastatic hepatocellular carcinoma.

## Introduction

Hepatocellular carcinoma (HCC) has a high probability of disease-related mortality.^1,2^ Due to the locally aggressive nature of HCC, more patients die from their intrahepatic disease burden and liver dysfunction compared with metastatic disease.^3,4^ The pattern of failure after both hepatectomy^5,6,7^ and other locoregional therapies (LRTs) is predominantly intrahepatic.^8,9,10,11,12^ Therefore, an in-depth understanding of the role of local, liver-directed therapy in the management of nonmetastatic HCC is critical to informing optimal management recommendations for patients with HCC.

In contrast to some cancers, several treatment options exist for nonmetastatic HCC. For patients who are operative candidates, surgical management with liver transplant or resection is preferred.^13,14,15^ For unresectable disease, LRT is often recommended.^16^ According to the National Comprehensive Cancer Network,^16^ LRT includes radiofrequency ablation (RFA) or microwave ablation (MWA), transarterial embolization methods (transarterial bland embolization [TAE], transarterial chemoembolization [TACE] with or without the used of drug-eluting beads [DEBs], or transarterial radioembolization [TARE]), and radiation therapy (RT), including stereotactic body RT, as well as transarterial hepatic infusion chemotherapy (HAIC),^17,18,19,20^ as detailed in other national guidelines.^21^ Systemic therapy alone is also an option for the treatment of unresectable HCC. This consists of tyrosine kinase inhibitors (TKIs)^22,23,24^ and newer immunotherapy-based regimens, including atezolizumab with bevacizumab^25^ and durvalumab with or without tremelimumab.^26^ How and when to use one intervention over—or in combination with—another, including systemic therapy, requires robust patient-centered and multidisciplinary discussion.

To our knowledge, there remains no consensus regarding the preferred treatment for nonmetastatic HCC when transplant is not an option. As such, clinicians lack high-level guidance on preferred management recommendations for patients not eligible for transplant. To address this knowledge gap, we conducted a systematic review of all randomized clinicals trials (RCTs) evaluating the treatment of HCC with LRT. We then performed meta-analyses to compare each LRT option with each other, pairwise. This presented not only an opportunity to define the variability between treatment regimens and protocols, but also to generate more-robust and generalizable summary estimates of relative treatment outcomes. Our aim was to examine the comparative outcomes of LRT-based treatment regimens and offer guidance to physicians in circumstances where multiple treatment options are viable.

## Methods

### Systematic Review

A computerized literature search and data extraction were performed by 2 investigators (H.M. and V.V.) independently. Databases searched included MEDLINE as well as the proceedings of the American Society of Clinical Oncology and American Society for Radiation Oncology annual meetings from January 1, 2010, to November 1, 2023, and the bibliographies of identified reports. The Population, Intervention, Control, Outcome, and Study Design framework was used to structure a search to select relevant phase 2 or 3 RCTs (eTable 1 in Supplement 1), along with the Preferred Reporting Items for Systematic Reviews and Meta-Analyses (PRISMA) reporting guideline. Core inclusion criteria were RCTs having investigated LRT for patients with nonmetastatic HCC. Single-arm and nonrandomized trials were excluded given the inability to determine comparative efficacy from these designs and the potential for bias in the assessment of this measure resulting from the heterogeneity in baseline covariates in trial populations. Further details regarding the search strategy, a full list of inclusion and exclusion criteria, and the data extraction method are reported in the eMethods in Supplement 1. A risk-of-bias assessment^27^ for each study was conducted as reported in eFigure 1 in Supplement 1.

### Statistical Analysis

The primary aim was to conduct meta-analyses to compare guideline-recommended LRTs (surgery with adjuvant LRT, surgery, RFA, MWA, RT, HAIC, TACE, TAE, and TARE) with one another and, subsequently, to profile the association between these treatments. First, each LRT was compared with all other available comparators to verify heterogeneity. We then estimated the pooled-effect estimates from each pairwise comparison and conducted subgroup analyses by comparator LRT subtype to examine whether heterogeneity was moderated by the comparator treatment. For our second aim, we compared each treatment class with others, pairwise (ie, systemic therapy, LRT, or the combination thereof), as detailed in the eAppendix in Supplement 1. Weighted random-effects meta-analyses were conducted given the anticipated heterogeneity between studies, and the DerSimonian and Laird method^28^ was used to obtain pooled estimates of progression-free survival (PFS) hazard ratios (HRs). Estimates of pooled overall survival (OS) HRs were also obtained, but our primary focus in this study was to produce a pooled estimate of the HRs for PFS, given the possibility of imbalanced postprotocol therapy between arms, which may influence OS.

Initially, we considered conducting a network meta-analysis.^29^ However, because we observed significant differences between treatment effect estimates attributed to indirect and direct evidence, we instead selected a direct, pairwise meta-analytic approach. For the pairwise meta-analysis, we used the HR as a measure of relative treatment effect within a trial. Because all studies included in this investigation were randomized, the experimental method of each study was anticipated to control for both known and unknown confounders, accounting for heterogeneity between arms within a study. Our focus on HRs derived from RCTs as a measure of comparative efficacy allowed for an estimate of treatment effect independent of the baseline risk of patients within a trial. Verifying this, we found a lack of evidence of any systematic difference in treatment effect by baseline trial population composition (parametrized by Child-Pugh score, surgical candidacy, viral-related cause of HCC, receipt of prior treatment, or estimated Barcelona Clinic Liver Cancer risk group^13^ [all analysis of variance *P* > .05]), further suggesting the viability of the analytic approach.

We used the Cochran *Q* test^30^ to assess heterogeneity, with *P* < .05 considered the threshold for significance. Subgroup analyses were used to identify homogeneous subgroups by treatment category when possible, including an exploratory subgroup analysis between HAIC agent defined by those containing fluorouracil vs those containing cisplatin. When the number of trial publications was adequate, we tested for the presence of publication bias with the Begg test^31^ and report findings when significant, along with a corrected HR using the trim-and-fill method.^32^ For each analysis, the hypothesis that an overall association of an intervention compared with the referent was tested via a 2-sided Wald test, with *P* < .05 considered significant. Given the exploratory nature of this investigation, no adjustments were made for multiple hypothesis testing. Data management and analyses were conducted using R, version 4.3.1 (R Project for Statistical Computing).

## Results

### Systematic Review

**Figure 1.**
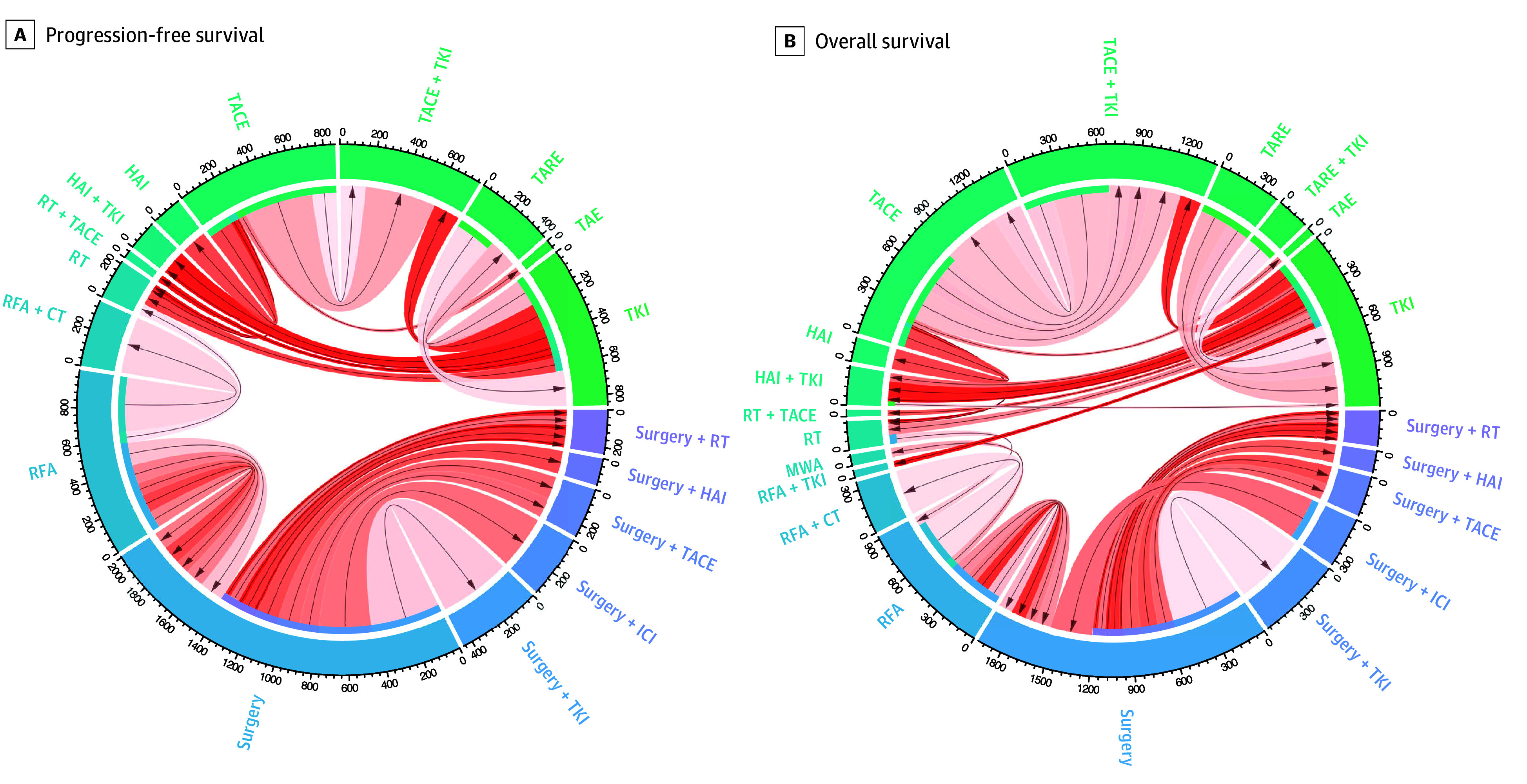
Size of Each Included Study This diagram represents each study included in the progression-free survival (30 studies, 8537 patients) (A) and overall survival (37 studies, 10 301 patients) (B) meta-analyses. Each chord represents a single study, with arrow direction and intensity of shading indicating the favored treatment and relative magnitude of treatment effect observed in that study. Arc lengths represent the number of patients treated with a specific therapy included in this analysis. CT indicates systemic therapy without specification of drug; HAI, hepatic arterial infusion; ICI, immune checkpoint inhibitor; MWA, microwave ablation; RFA, radiofrequency ablation; RT, radiotherapy-based treatment; TACE, transarterial chemoembolization; TAE, transarterial bland embolization; TARE, transarterial radioembolization; and TKI, tyrosine kinase inhibitor.

The search method identified 1915 MEDLINE records and 1488 abstracts, of which 57 reports were retained for full-text review. After the prespecified exclusion criteria were applied, 40 trials reporting on 11 576 patients were included in this analysis, with PFS evaluated in 30 studies (n = 8537 patients) and OS in 37 studies (n = 10 301 patients) (eFigure 2 in Supplement 1). The median trial follow-up was 30.0 (IQR, 18.5-40.8) months for those reported. The baseline characteristics of the studies are detailed in eTable 2 in Supplement 1. The corresponding network of comparisons is summarized in Figure 1 and the studies are further detailed in eTable 3 in Supplement 1.

### LRT Comparison

#### Surgical Management

**Figure 2.**
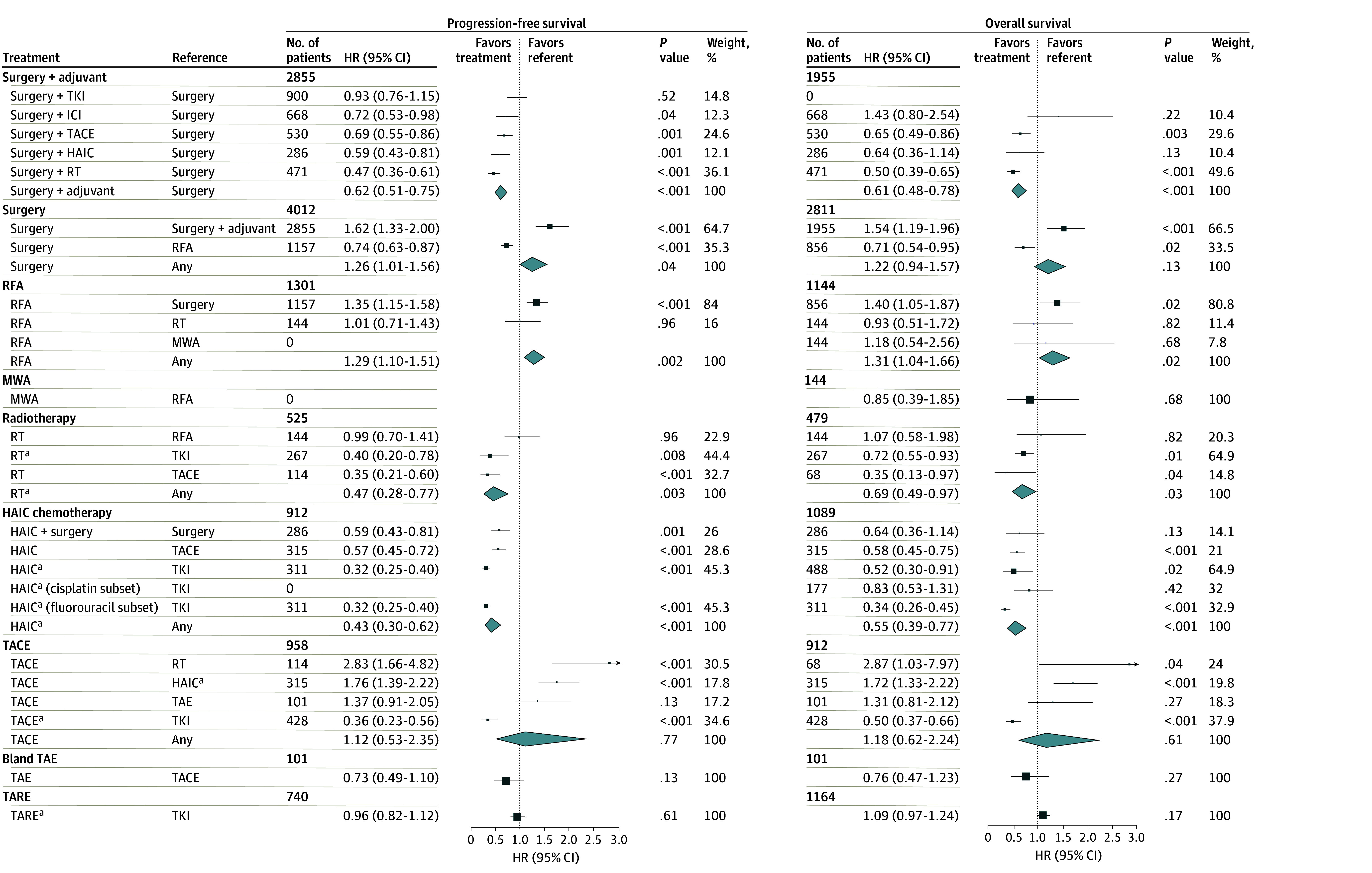
Trial-Level Relative Treatment Effects From Each Profiled Randomized Clinical Trial Box areas are proportional to weights in each meta-analysis, and diamond widths are proportional to the precision of the pooled estimate from a given meta-analysis. HAIC indicates hepatic arterial infusion chemotherapy; HR, hazard ratio; ICI, immune checkpoint inhibitor; MWA, microwave ablation; RFA, radiofrequency ablation; RT, radiotherapy; TACE, transarterial chemoembolization; TAE, transarterial bland embolization; TARE, transarterial radioembolization; and TKI, tyrosine kinase inhibitor. ^a^Pooled outcome with or without the addition of adjunctive therapy.

Overall, we identified 15 RCTs^7,33,34,35,36,37,38,39,40,41,42,43,44,45,46^ representing 4012 patients studying partial hepatectomy. The patient populations included in these studies were unique among those analyzed, as they uniformly required patients to be eligible for surgery. We found 10 studies that compared the efficacy of intensification of surgery with adjuvant therapy (RT,^33,34,35,36,37^ HAIC,^38^ TACE,^7,39^ atezolizumab with bevacizumab,^40^ and TKI^41^) with surgery alone (ie, partial hepatectomy). Together, adjuvant treatment was associated with improved PFS (HR, 0.62 [95% CI, 0.51-0.75]; *P* < .001) and OS (HR, 0.61 [95% CI, 0.48-0.78]; *P* < .001) (Figure 2; eFigure 3 in Supplement 1). A test for publication bias suggested the possibility of incomplete reporting for the PFS end point of the surgery with or without adjuvant therapy analysis (*z* = −2.59; *P* = .01) and a corrected PFS HR was estimated to be 0.76 (95% CI, 0.63-0.94; *P* = .01) using the trim-and-fill method. In an additional 5 studies that compared surgery with RFA and were without evidence of heterogeneity,^42,43,44,45,46^ surgery was associated with improved outcomes over RFA for both PFS (HR, 0.74 [95% CI, 0.63-0.87]; *P* < .001) and OS (HR, 0.71 [95% CI, 0.54-0.95]; *P* = .02) (Figure 2; eFigure 4 in Supplement 1).

#### RFA and MWA

Seven studies containing data from 1445 patients involving RFA were identified.^42,43,44,45,46,47,48^ In addition to the 5 studies comparing RFA and surgery,^42,43,44,45,46^ 1 trial compared RFA with MWA^47^ (4- to 6-minute application at 140 W) and the other with 66 GyE in 10 fractions via proton beam therapy.^48^ Neither trial observed evidence of a significant difference between RFA and MWA (OS: HR, 1.18 [95% CI, 0.54-2.56]; *P* = .68) or RFA and RT (PFS: HR, 1.01 [95% CI, 0.71-1.43]; *P* = .96; OS: HR, 0.93 [95% CI, 0.51-1.72]; *P* = .82) in the end points reported. These results are summarized in Figure 2.

#### Radiotherapy

Six studies^48,49,50,51,52,53^ were pooled comparing RT-containing treatment with other therapies, representing 555 patients. The pooled HRs favored RT over the referent arms on the end points of PFS (HR, 0.47 [95% CI, 0.28-0.77]; *P* = .003) and OS (HR, 0.69 [95% CI, 0.49-0.97]; *P* = .03) (Figure 2). This was attributable to the contribution of the subgroup comparisons of RT with TKI (PFS: HR, 0.40 [95% CI, 0.20-0.79]; *P* = .008; OS: HR, 0.72 [95% CI, 0.55-0.93]; *P* = .01), and RT with TACE (PFS: HR, 0.35 [95% CI, 1 0.21-0.60]; *P* < .001; OS: HR, 0.35 [95% CI, 0.13-0.97]; *P* = .04) (eFigure 5 in Supplement 1).

#### Hepatic Arterial Infusion Chemotherapy 

We identified 6 trials containing 1089 total patients that used cisplatin-based^18,54^ or fluorouracil-based^17,19,38,55^ HAIC. The trials compared HAIC with sorafenib alone^17,18,19,54^ or TACE,^55^ or profiled its use as a surgical adjuvant.^38^ Globally, the pooled treatment effect estimates for both PFS (HR, 0.43 [95% CI, 0.30-0.62]; *P* < .001) and OS (HR, 0.55 [95% CI, 0.39-0.77]; *P* < .001) suggested an association with better outcomes for HAIC-containing arms (Figure 2). Hepatic arterial infusion chemotherapy was associated with better outcomes over TACE (PFS: HR, 0.57 [95% CI, 0.45-0.72]; *P* < .001; OS: HR, 0.58 [95% CI, 0.45-0.75]; *P* < .001). Despite evidence of significant subgroup differences between referent treatments for the PFS analysis (*Q*_2_ = 14.66; *P* < .001) not present in the OS analysis (*Q*_2_ = 0.26; *P* = .88), there was consistent evidence that treatments that included HAIC were associated with improved outcomes. In a subgroup analysis of all studies that included treatment with HAIC by HAIC agent, OS associated with HAIC was restricted to studies with fluorouracil-based HAIC (fluorouracil: HR, 0.45 [95% CI, 0.31-0.64]; *P* < .001; cisplatin: HR, 0.83 [95% CI, 0.53-1.31]; *P* = .42) (eFigure 6 in Supplement 1). Data availability limited a similar analysis for PFS.

#### Embolization-Based Therapies: TACE, TAE, and TARE

Seven studies containing 988 patients were identified comparing TACE-containing therapy with 4 other treatment modalities: RT,^49,50,52^ as discussed in the RT subsection; HAIC,^55^ as discussed in the HAIC subsection; bland TAE^56^; and TKI^53,57^ (Figure 2). We detected significant heterogeneity in treatment effect between studies when analyzing both PFS and OS (both *P* < .001) that resolved on subgroup analysis (eFigure 7 in Supplement 1). TACE was observed to be associated with worse outcomes compared with RT and HAIC on both PFS and OS. TACE appeared similar to bland TAE based on a single study^56^ (PFS: HR, 1.37 [95% CI, 0.91-2.05]; *P* = .13; OS: HR, 1.31 [95% CI, 0.81-2.12]; *P* = .27), and TACE-based treatment appeared to be associated with better outcomes than TKI monotherapy, based on 2 trials^53,57^ consisting of 428 patients (PFS: HR, 0.36 [95% CI, 0.23-0.56]; *P* < .001; OS: HR, 0.50 [95% CI, 0.37-0.66]; *P* < .001). Three studies^58,59,60^ without evidence of significant heterogeneity in comparing TARE-based treatment with TKI were identified, and together, the pooled treatment effect estimate of these studies indicated no significant difference between TARE and TKI therapy in PFS or OS (eFigure 8 in Supplement 1).

#### Comparison of All Treatment Arms

**Figure 3.**
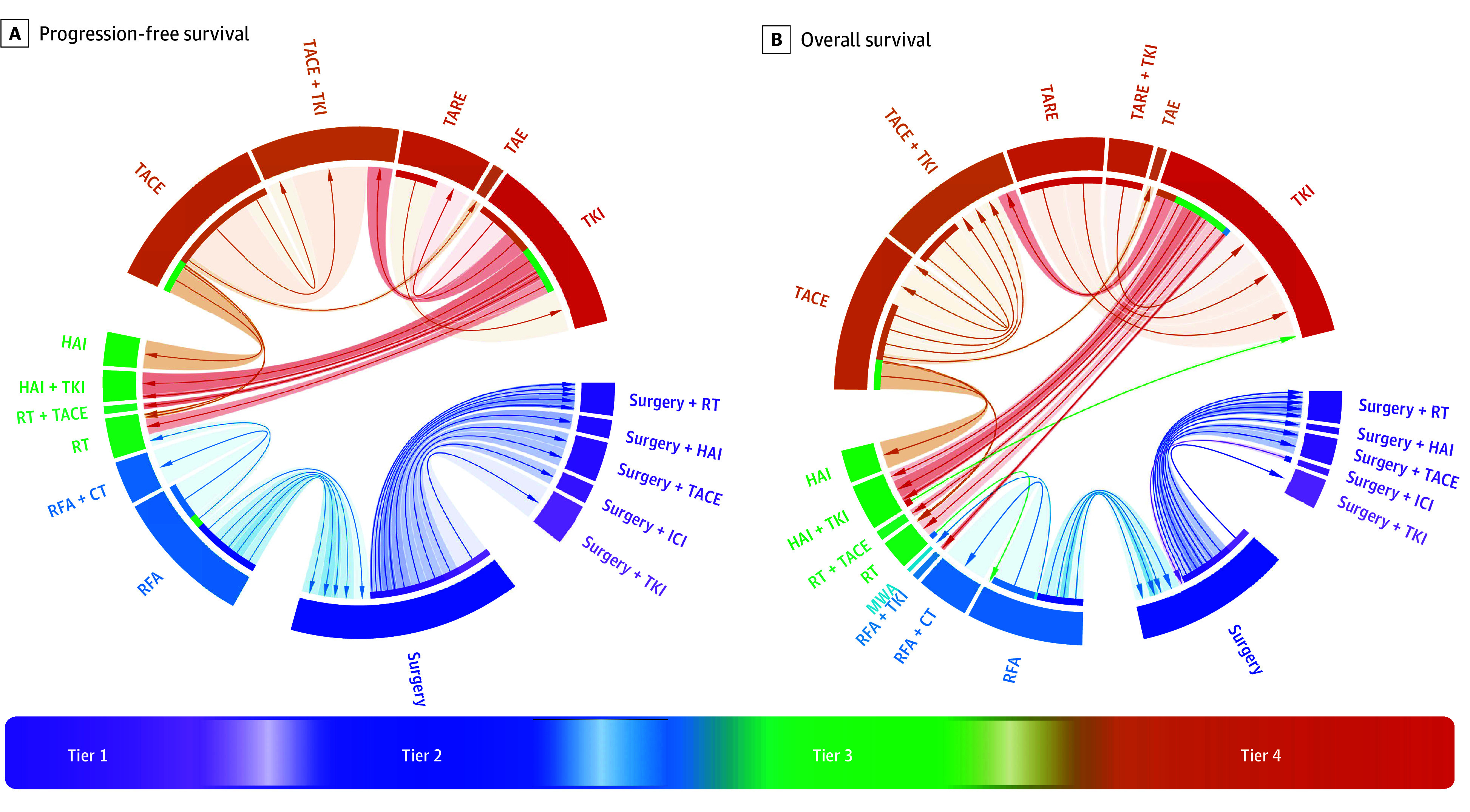
Treatment Effect Estimates of Each Profiled Locoregional Therapy The relative treatment effect (ie, hazard ratio) from each trial is included in this analysis and shows the emergence of a 4-tier ordinal structure of locoregional therapies (LRTs) at the trial level, where treatment effects favor LRTs either within the same tier or in a lower-numbered tier (ie, tier 1 > 2 > 3 > 4), and this is congruent with the findings from the pairwise meta-analysis. Arrows point to the LRT with a more favorable outcome in a randomized comparison, and shading intensity is proportional to the magnitude of the treatment effect. Arc lengths are proportional to the amount of information informing a specific trial comparison. CT indicates chemotherapy; HAI, hepatic arterial infusion; ICI, immune checkpoint inhibitor; MWA, microwave ablation; RFA, radiofrequency ablation; RT, radiotherapy-based treatment; TACE, transarterial chemoembolization; TAE, transarterial bland embolization; TARE, transarterial radioembolization; and TKI, tyrosine kinase inhibitor.

Each treatment was compared pairwise at the trial level, and we observed similar hierarchy of relative treatment effects consistent with those identified by the pooled analysis (Figure 3). This suggested a 4-tier structure: (1) partial hepatectomy with and (2) without adjuvant therapy; (3) LRT with RFA, RT, or HAIC; and (4) TACE-, TARE-, and TAE-based LRT or TKI monotherapy. Directionally, all treatment effects were associated with treatments either within the same tier or in a lower numbered tier (ie, tier 1 > 2 > 3 > 4). We observed 2 exceptions in this directionality for the IMbrave050^40^ OS analysis, which compared surgery with or without atezolizumab plus bevacizumab, and SCOOP-2,^18^ which compared sorafenib with or without cisplatin HAIC, possibly due to the low precision in the point estimate of treatment effect arising from low event rates during the reported follow-up period. Statistically significant differences in treatment effect were more common between tiers than within tiers (PFS: χ^2^_1_ = 3.75; *P* = .03; OS: χ^2^_1_ = 4.90; *P* = .01), and treatment effects were consistently greater in trials that compared treatments across tiers as opposed to within tiers (PFS: *t* = −3.65; *P* = .003; OS: *t* = −4.79; *P* < .001). In addition, we sought to examine the ordinality in treatment effect suggested by the pairwise meta-analyses. To reiterate, we did not select network meta-analysis as our primary analytic method because of the observed differences in direct and indirect estimates of treatment effect. Nevertheless, the ordinality of treatments using a network meta-analysis was very similar to our pairwise meta-analysis method (eFigure 9 in Supplement 1). Minor deviations were observed again in cases where estimated treatment effects were based on low numbers of events resulting in low precision.

## Discussion

In this systematic review and pairwise meta-analysis of RCTs, we compared the efficacy of various LRTs in patients with localized HCC and provide pooled estimates of treatment effect (ie, HRs) for each comparison of all identified RCTs. Based on this analysis, we found that a hierarchical structure emerges between LRTs that is observed both on end points of PFS and OS. As the most significant pattern of failure in the transplant-ineligible population is local,^7,61^ it is not unexpected that the form of LRT may be associated with PFS if each LRT is nonequivalent. Given that progression is often fatal for patients with HCC, this signal may be similarly observed in the measurement of OS, although the similarity between these 2 may also be a result of the high competing risks in this population. The importance of LRT use and type is supported by our primary and supplemental analyses.

We found that surgical management represents an evidence-based, preferred, standard of care for patients with nonmetastatic HCC eligible for surgery. Adjuvant treatment may augment this standard, improving PFS and OS. Furthermore, we found that RFA appeared to be associated with worse outcomes compared with surgery, yet similar to the other available comparators (MWA^47^ and RT^48^). While the single trial comparing MWA to RFA^47^ did not find a statistically significant difference in PFS or OS, it was underpowered for the assessment of equivalence. In contrast, the study that compared RT with RFA^48^ found RT to be noninferior to RFA based on the primary end point: local PFS.

Radiotherapy- and HAIC-based treatment appeared to improve PFS and OS compared with either TACE or TKI monotherapy, and these findings were consistent with a recent meta-analysis observing that HAIC-containing treatments appeared to be superior to non–HAIC-containing treatments.^62^ As is true of all findings of the present study, these findings must be interpreted in the context of the heterogeneity of techniques and comparisons within a treatment category. Prior work has suggested that technical details of RT, such as dose and fractionation, may play an integral role in determining efficacy.^12,63^ A formal analysis of this could not be performed due to incomplete reporting and uncertainties regarding dose equivalency between various fractionation schedules. In contrast, a subgroup analysis of HAIC was possible, suggesting that fluorouracil-based HAIC was associated with better survival compared with the comparator condition, although similar results were not shown for cisplatin-based HAIC.

Next, we evaluated embolization-based treatments (TACE with or without DEB, TARE, and TAE), and trials using TACE with or without DEBs were pooled given randomized evidence of similar efficacy.^64^ These embolization-based methods were associated with worse outcomes compared with RT and HAIC. Although TARE appeared similar to TKI monotherapy, TACE appeared better than TKI monotherapy. No RCTs directly compared TACE with TARE, although a prospective nonrandomized study^65^ suggested that TARE may have an improved time-to-progression and a similar OS compared with TACE.

The trial-level data suggested an ordinal, 4-tier system for both the PFS and OS analyses. These tiers verified and recapitulated the described pooled findings. Surgical-based treatments (tiers 1 and 2) appear to be associated with better outcomes than other LRTs as indicated by the direction of the arrows in Figure 3. Adjuvant treatment was also associated with improved outcomes after surgery (tier 1 > 2). Nonembolization-based treatments (tier 3: RFA, MWA, RT, and HAI) outperformed embolization-based treatments (tier 4: TAE, TACE with or without DEB, and TARE), which themselves appear to perform similarly to TKI monotherapy. Accounting for uncertainty in the ordinality of low-precision estimates, a network meta-analysis also recapitulated these findings. While caution is warranted in using the resulting treatment effect estimates, the ordinal consistency with the primary analysis supports the findings of this study. Future studies will be important to address the role of immunotherapy in combination with LRT as under study in several trials,^66,67,68,69^ pending mature results.

### Limitations

This study has several limitations, including those related to the analytic method and those related to the underlying trial data, particularly a lack of individual patient-level data. In principle, a meta-analysis is meant to pool the available evidence to estimate the population-level treatment effect estimate for a specific parameter. Observed heterogeneity between trial-level observations limit this in practice, which comes in 2 forms: statistical- and design-related heterogeneity. Statistical heterogeneity is omnipresent and a result of pooling individual trials, which themselves only study a sample of the population of interest, and the meta-analytic framework used herein is a validated means by which to deal with this source of heterogeneity.

Design-level heterogeneity may arise when differences exist between studies in population or treatment. Herein, our systematic review was limited to high-quality RCTs, which, by their design, control for both known and unknown confounders, allowing for the estimate of treatment effect to be unbiased and maximally account for heterogeneity between treatment arms within an RCT. We used HR as the basis for this meta-analysis as it is a measure of relative treatment effect independent of the baseline hazard or baseline risk profile of the trial population. This allows for the pooling of trials with populations at various levels of baseline risk, therefore minimizing the influence of design-level heterogeneity; however, subtle differences in the quality of similarly labeled treatments (eg, RFA ablation zone or RT dose) may still contribute to design-level heterogeneity. Given the differences in trial population composition and baseline risk, we also avoided the more common network meta-analysis in favor of direct, pairwise meta-analyses in this report. This strategy requires fewer assumptions and is less vulnerable to differences in populations between studies. Nevertheless, design-level heterogeneity may theoretically represent a strength in interpretation, allowing for more general conclusions to be made, even when implementations of a treatment or included study populations may be slightly heterogeneous (eg, RFA as a concept as opposed to 500 kHz RFA for <2-cm tumors). We used random-effects meta-analyses to account for the anticipated design-level heterogeneity.

Despite methodologic rigor, residual limitations of the underlying trial data exist. While the inclusion criteria for this study were purposefully limited to RCTs, residual confounding may have impacted observed effect estimates in smaller trials. Additional limitations include protocol deviations within each trial, differences in the PFS definition, and differences in postprotocol therapy, which may affect the OS end point.

## Conclusions

The results of this systematic review and pairwise meta-analysis suggest that, of the LRTs profiled, some may produce different outcomes for patients with localized HCC compared with others. In this study, we found surgical-based management plans to be associated with the best outcomes with the possibility that adjuvant therapy may improve on these outcomes for select patients. Additional work will be required to examine which patients are optimal candidates for adjuvant treatment. Furthermore, the body of evidence suggests a difference between the remaining nonsurgical LRTs. Nonembolization-based treatments (RFA, MWA, RT, and HAIC) appeared to outperform embolization-based procedures (TAE, TACE with or without DEB, and TARE), which, themselves, appeared to perform similarly to TKI monotherapy.

In aggregate, our findings should be regarded as hypothesis-generating, requiring further verification in multi-arm randomized trials or via individual patient-level meta-analyses. These findings underscore the need for a multidisciplinary, individualized treatment approach for all patients with nonmetastatic HCC.

## Notes

Supplement 1. **eMethods.** Detailed Methods
eReferences
**eTable 1.** PICOS Framework Summary **eTable 2.** Baseline Characteristics of Evaluable Studies **eTable 3.** Select Outcomes of Evaluable Studies **eFigure 1.** Risk of Bias Assessment **eFigure 2.** PRISMA Flowchart of Study Selection **eFigure 3.** Surgery ± Adjuvant **eFigure 4.** Surgery vs RFA **eFigure 5.** RT vs Other **eFigure 6.** HAI vs Other **eFigure 7.** TACE vs Other **eFigure 8.** TARE vs TKI-Based Therapy **eFigure 9.** Network Meta-Analysis
eAppendix

Supplement 2.
Data Sharing Statement
