# Supplementary material for: Locoregional Therapies for Hepatocellular Carcinoma: A Systematic Review and Meta-Analysis
Source: JAMA Netw Open. 2024 Nov 27;7(11):e2447995. doi: 10.1001/jamanetworkopen.2024.47995 (PMC12527482; doi:10.1001/jamanetworkopen.2024.47995)
Supplement: Supplement 1. — eMethods. Detailed Methods eReferences eTable 1. PICOS Framework Summary eTable 2. Baseline Characteristics of Evaluable Studies eTable 3. Select Outcomes of Evaluable Studies eFigure 1. Risk of Bias Assessment eFigure 2. PRISMA Flowchart of Study Selection eFigure 3. Surgery ± Adjuvant eFigure 4. Surgery vs RFA eFigure 5. RT vs Other eFigure 6. HAI vs Other eFigure 7. TACE vs Other eFigure 8. TARE vs TKI-Based Therapy eFigure 9. Network Meta-Analysis eAppendix [file jamanetwopen-e2447995-s001.pdf]

Supplementary Online Content

Patel KR, Menon H, Patel RR, Huang EP, Verma V, Escorcia FE. Locoregional therapies for hepatocellular carcinoma: a systematic review and meta-analysis. *JAMA Netw Open*. 2024;7(11):e2447995. doi:10.1001/jamanetworkopen.2024.47995

eMethods. Detailed Methods
eReferences
eTable 1. PICOS Framework Summary
eTable 2. Baseline Characteristics of Evaluable Studies
eTable 3. Select Outcomes of Evaluable Studies
eFigure 1. Risk of Bias Assessment
eFigure 2. PRISMA Flowchart of Study Selection
eFigure 3. Surgery ± Adjuvant
eFigure 4. Surgery vs RFA
eFigure 5. RT vs Other
eFigure 6. HAI vs Other
eFigure 7. TACE vs Other
eFigure 8. TARE vs TKI-Based Therapy
eFigure 9. Network Meta-Analysis
eAppendix

This supplementary material has been provided by the authors to give readers additional information about their work.

© 2024 Patel KR et al. *JAMA Network Open.*

eMethods. Detailed Methods

The Population, Intervention, Comparison, Outcomes, and Study (PICOS) framework was used to structure the initial search strategy (**eTable 1**), and the methodology for the subsequent systematic review reporting followed the Preferred Reporting Items for Systematic Reviews and Meta-Analyses (PRISMA). A systematic literature review was performed in PubMed (MEDLINE) as well as the proceedings of the American Society of Clinical Oncology (ASCO) and American Society for Radiation Oncology (ASTRO) annual meetings. We reviewed the reference lists of included publications along with relevant review articles retrieved from the electronic searches to identify other potentially relevant studies that could have been missed. The initial search strategy was as follows: "hepatic" OR "liver" AND "carcinoma" OR "cancer" OR "malignancy" OR "neoplasm" AND "prospective" OR "randomized" OR "trial". Searches were conducted by multiple authors and included reports published in the English language before November 1, 2023. If a trial had been updated, the publication with the most updated data was included. Conflicts were resolved via direct discussion between the authors.

Studies were eligible if they met the following inclusion criteria: (1) patient population representing nonmetastatic HCC (studies with < 5% of patients with metastases were included as these studies were felt to measure the relevant population of interest), (2) randomized phase II or phase III trials with protocol-mandated surgical management, locoregional therapy (LRT) as defined by the NCCN[1] or other national guidelines[2] (RFA, MWA, TAE, [DEB-]TACE, TARE, RT, HAIC) in at least one arm (including studies of adjuvant treatment), and (3) reported data sufficient for quantitative meta-analysis for PFS, OS, or both. Exclusion criteria were as follows: (1) studies comparing variants of the same class of therapy, (2) publications involving a placebo control comparator, (3) trials with planned subtotal and/or incomplete locoregional treatment, (4) reports of incomplete studies or those which were assessed as having high risk of bias identified by the study investigators, and (5) data reported solely in meta-analyses, reviews, surveys, press releases, letters, and book chapters. Each study was assessed for bias using the Cochrane RoB2 tool[3] as shown in **eFigure 1**.

Data Extraction

From each study, extracted data included the first author's name, study year, country of enrollment, baseline trial cohort composition, trial design, treatment regimens, number of patients, as well as a measure of treatment effect for the comparison (i.e., hazard ratios [HR]) for overall survival (OS) and progression free survival (PFS). When HRs were not reported and Kaplan-Meier curves with corresponding risk tables were reported, WebPlotDigitizer version 4.6 was used to extract survival data and estimate the HR. When PFS was not reported, composite endpoints of survival and disease recurrence were utilized for the PFS endpoint. As this study focused exclusively on randomized trials, unadjusted, intention-to-treat analyses were utilized.

© 2024 Patel KR et al. *JAMA Network Open.*

eReferences

1. Network NCC. Hepatocellular Carcinoma (Version 2.2023). 2023. Accessed November 22, 2023, 2023. https://www.nccn.org/professionals/physician_gls/pdf/hcc.pdf
2. Kudo M, Kawamura Y, Hasegawa K, et al. Management of Hepatocellular Carcinoma in Japan: JSH Consensus Statements and Recommendations 2021 Update. *Liver cancer*. Jun 2021;10(3):181-223. doi:10.1159/000514174
3. Higgins JPT, Altman DG, Gøtzsche PC, et al. The Cochrane Collaboration's tool for assessing risk of bias in randomised trials. *BMJ*. 2011;343:d5928. doi:10.1136/bmj.d5928

© 2024 Patel KR et al. *JAMA Network Open.*

eTable 1. PICOS Framework Summary

PICOS Framework Utilized for Analysis 1	
**Participants**	Nonmetastatic hepatocellular carcinoma ineligible for liver transplant*
**Interventions**	Any locoregional therapy for treatment of HCC:<br>1. surgery without adjuvant therapy,<br>2. surgery with adjuvant therapy,<br>3. radiofrequency ablation (RFA),<br>4. microwave ablation (MWA),<br>5. radiotherapy (RT),<br>6. hepatic arterial infusion (HAI) chemotherapy,<br>7. transarterial chemoembolization (TACE),<br>8. transarterial radioembolization (TARE),<br>9. bland embolization (TAE),
**Comparisons**	Prospective randomized comparisons of any locoregional therapy compared against another locoregional therapy or a systemic therapy option
**Outcomes**	Progression Free Survival (PFS) and/or Overall Survival (OS)
**Study design**	Randomized, controlled phase II or III trials
**PICOS Framework Utilized for Analysis 2**	
**Participants**	Nonmetastatic hepatocellular carcinoma ineligible for liver transplant*
**Interventions**	1. Locoregional therapy alone<br>2. Systemic therapy alone<br>3. A protocol treatment utilizing a combination of locoregional therapy and systemic therapy
**Comparisons**	Prospective randomized comparisons of any of the above interventions or combinations thereof
**Outcomes**	Progression Free Survival (PFS) and/or Overall Survival (OS)
**Study design**	Randomized, controlled phase II or III trials
* Trials with < 5% metastatic patients will be included as it is felt that such trials will meaningfully represent the sought after effect estimates	

© 2024 Patel KR et al. *JAMA Network Open.*

eTable 2. Baseline Characteristics of Evaluable Studies

Study	Country	Patient per arm (n)	Median Age	Sex by Arm (M/F)	Tumor Size	Child-Pugh Score	Viral Hepatitis Etiology	Serum AFP (ng/mL)	Surgical Candidate	Prior Treatment
Ng et al. 2017	China	109, 109	55; 57	89/20; 86/23	< 4 cm	A	Any	58; 63.5	Yes	None
Xia et al. 2020	China	120, 120	50; 50	107/13; 109/11	< 4 cm, number < 3	A	Any	70; 73	Yes	Partial Hepatectomy
Feng et al. 2012	China	84, 84	51; 47	79/5; 75/9	< 4 cm, number < 3	A or B	HBV	215.5; 262.8	Yes	None
Huang et al. 2010	China	115, 115	55.9; 56.7	85/30; 79/36	< 5 cm, number < 3	A or B	HBV	NR	Yes	None
Takamaya et al. 2022	Japan	151, 150	68; 69	112/38; 108/43	< 3 cm, number < 3	A	Any	NR	Yes	None
Comito et al. 2022	Italy	21. 19	75; 75	15/6; 15/4	BCLC Stage A/B	A or B	Any	NR	No	Prior TACE
Romero et al. 2023	Multinational	12, 16	69; 62	10/2; 14/2	< 6 cm total, number < 3	A	Any	8; 5	No	None
Kim et al. 2021	Korea	72, 72	60; 61.5	61/11; 59/13	< 3 cm, number < 2	A	HBV	4.9; 5.1	No	Prior resections permitted
Bush et al. 2023	United States	35, 39	61.7; 59.6	27/8; 26/13	< 5 cm, number < 3	A or B	Any	NR	No	None

© 2024 Patel KR et al. *JAMA Network Open.*

Yoon et al. 2018	Korea	45, 45	55; 55	77/13; 39/6	1 measurable lesion	A	Any	667; 1496	No	None
Chow et al. 2018	Multinational	182, 178	59.5; 57.7	147/35; 151/27	BCLC Stage B/C	A or B	Any	NR	No	< 2 prior Hepatic/ directed therapies permitte d
Vilgrain et al. 2017	France	174, 206	66;65	212/25; 202/20	BCLC Stage C	A or B	Any	87;80	No	Prior resection s permitte d
Zheng et al. 2022	China	32, 32	56;55	30/2; 31/1	inoperable HCC	A	Any	310; 655	No	No prior HAI or systemic therapy
He et al. 2019	China	125, 122	49; 49	111/14; 112/10	inoperable HCC	A	Any	5922; 6666	No	None
Ikeda et al. 2016	Japan	66, 42	64; 66	56/9; 32/9	inoperable HCC	A or B	Any	188; 223.5	No	No prior HAI or systemic therapy
Kondo et al. 2019	Japan	36, 34	70.9; 72	27/6; 28/7	Any HCC deemed to have limited benefit from surgery	A or B	Any	216; 67.3	No	All prior treatmen t permitte d, 4/week washout
Giorgio et al. 2016	Italy	49, 50	71;72	37/12; 36/14	< 5 cm, number < 3, with PVTT	A	Any	83;80	No	None
Li et al. 2021	China	159, 156	53; 54	134/24; 141/15	BCLC Stage A/B	A	Any	NR	Yes	None

© 2024 Patel KR et al. *JAMA Network Open.*

Peng et al. 2023	China	170, 168	54; 56	139/31; 132/36	Locally advanced	A	Any	55,979; 31,752	No	Prior resections permitted
Dawson et al. 2022	United States	85, 92	66	NR	BCLC Stage B/C	A	Any	NR	No	Prior resections permitted
Ricke et al. 2019	Multinational	216, 208	66; 66	181/31; 177/30	BCLC Stage A/C	A or B	Any	NR	No	Prior resections or liver/directed therapies (>3 months) permitted
Qin et al. 2023	Multinational	334, 334	62; 60	289/53, 281/51	BCLC Stage B/C	A or B	Any	NR	No	Prior curative treatment
Kudo et al. 2011	Multinational	229, 229	69; 70	174/55; 160/69	< 10 lesions; < 70 mm in greatest dimension	NR	Any	NR	No	Prior local therapy
Kudo et al. 2018	Multinational	444, 444	66.2; 65.4	363/81; 364/80	BCLC Stage A/C	A	Any	NR	No	Prior Local therapy
Kudo et al. 2014	Multinational	249, 253	57;59	206/43; 216/37	< 4 lesions, one measured >5 cm	B or C	Any	NR	No	No prior treatment
Tak et al. 2018	Multinational	354, 347	NR	267/87; 263/84	BCLC Stage A/B	A or B	Any	NR	No	No prior treatment
Bruix et al. 2015	Multinational	556, 558	58;60	451/105; 461/97	Single lesion < 5 cm or 3 lesions < 3 cm	A or B	Any	6; 5.6	Yes	Prior resection or ablation

© 2024 Patel KR et al. *JAMA Network Open.*

Lencioni et al. 2016	Multinational	154. 153	64.5; 63	135/19; 126/27	BCLC Stage B	A	Any	NR	No	No prior local therapy
Meyer et al. 2017	United Kingdom	157. 156	65; 68	139/18; 138/18	1 measurable lesion	A	Any	23; 25	No	No prior local therapy
Brown et al. 2016	United States	51, 50	68; 65	37/14; 41/9	Okuda Stage I or II	A or B	Any	NR	No	NR
Wang et al. 2018	China	140, 140	52.6, 54.2	109/31; 121/19	NR	A or B	HBV	NR	Yes	No prior treatmen t
Li et al. 2023	China	143, 143	50; 54	136/21; 139/19	NR	A or B	Any	164; 189	N/A	Prior resection
Sun et al. 2019	China	26, 26	49.6; 51.1	24/2; 24/2	BCLC Stage A or B	A	HBV	NR	N/A	Prior resection
Li et al. 2020	China	78, 78	53.0; 53.0	58/20; 61/17	NR	A	Any	27.7; 50.2	N/A	Prior resection
Chen et al. 2013	China	34, 34	48.9; 50.8	24/10; 25/9	BCLC Stage A or B	A	Any	579; 612	N/A	Prior resection
Rong et al. 2020	China	61, 58	53.1; 55.5	44/1; 31/9	BCLC Stage 0 or A	NR	Any	NR	Yes	No prior treatmen t
Shi et al. 2022	China	38. 38	56.2; 55.7	33/5; 36/2	BCLC Stage 0 or A	NR	Any	NR	N/A	Prior Resectio n
Wei et al. 2018	China	125, 125	44, 48.5	106/10; 106/12	BCLC Stage A	A or B	Any	NR	N/A	Prior resection
Vietti Violi et al. 2018	Multinational	73, 71	68; 65	59/12; 62/11	< 3 lesions all < 3 cm in diameter	A or B	Any	NR	No	No prior treatmen t

Abbreviations: HCC = Hepatocellular Carcinoma, BCLC = Barcelona Cancer Liver Clinic, TACE = transarterial chemoembolization, HAI = hepatic artery infusion. NR = not reported.

© 2024 Patel KR et al. *JAMA Network Open.*

eTable 3. Select Outcomes of Evaluable Studies

Study	Arm 1	Arm 2	Median Follow-Up (months)	Primary Outcome	Included in Analysis 1 PFS	Included in Analysis 1 OS	Included in Analysis 2 PFS	Included in Analysis 2 OS
**Ng et al. 2017**	RFA	Surgery	93	Tumor recurrence	No	No	Yes	Yes
**Xia et al. 2020**	RFA	Surgery	44.3	OS	No	No	Yes	Yes
**Feng et al. 2012**	RFA	Surgery	36	OS	No	No	Yes	Yes
**Huang et al. 2010**	RFA	Surgery	37.1, 46.4	OS	No	No	Yes	Yes
**Takamaya et al. 2022**	RFA	Surgery	59.9, 60.5	RFS	No	No	Yes	No
**Comito et al. 2022**	TACE	RT (30-75 Gy in 3-10 fx)	20	LC	No	No	Yes	Yes
**Romero et al. 2023**	TACE-DEB	SBRT (54 Gy in 3 fractions)	28.1	TTP	No	No	No	Yes
**Kim et al. 2021**	PBT (66 Gy in 10 fractions)	RFA	51.6, 50.7	LPFS	No	No	Yes	Yes
**Bush et al. 2023**	PBT (70.2 Gy in 15 fractions)	TACE	30	OS	No	No	Yes	No
**Yoon et al. 2018**	Sorafenib	TACE + EBRT (45 Gy in 15-18 fractions)	35	PFS	Yes	Yes	Yes	Yes
**Chow et al. 2018**	Sorafenib	TARE	NR	OS	Yes	Yes	Yes	Yes
**Vilgrain et al. 2017**	Sorafenib	TARE	25.7, 30.1	OS	Yes	Yes	Yes	Yes
**Zheng et al. 2022**	Sorafenib	Sorafenib + 5FU/Oxaliplatin HAI	25.0, 16.4	OS	Yes	Yes	Yes	Yes

© 2024 Patel KR et al. *JAMA Network Open.*

**He et al. 2019**	Sorafenib	Sorafenib + FOLFOX HAI	28	OS	Yes	Yes	Yes	Yes
**Ikeda et al. 2016**	Sorafenib	Sorafenib + CDDP	NR	OS	No	Yes	No	Yes
**Kondo et al. 2019**	Sorafenib	CDDP HAI + Sorafenib	NR	OS	No	Yes	No	Yes
**Giorgio et al. 2016**	Sorafenib	Sorafenib + RFA	NR	OS	No	Yes	No	No
**Li et al. 2021**	TACE	FOLFOX HAI	NR	OS	No	No	Yes	Yes
**Peng et al. 2023**	Lenvatinib	TACE + Lenvatinib	17	OS	Yes	Yes	Yes	Yes
**Dawson et al. 2022**	Sorafenib	Sorafenib + SBRT	13.2	OS	Yes	Yes	Yes	Yes
**Ricke et al. 2019**	Sorafenib	TARE + Sorafenib	9.4, 6.6	OS	No	Yes	No	Yes
**Qin et al. 2023**	Surgery	Surgery + Atezolizumab + Bevacizumab	33	OS	Yes	Yes	Yes	Yes
**Kudo et al. 2011**	TACE + Sorafenib	TACE	NR	TTP	No	Yes	No	No
**Kudo et al. 2018**	TACE + Orantinib	TACE	NR	OS	Yes	Yes	No	No
**Kudo et al. 2014**	TACE + Brivanib	TACE	16.6	OS	No	Yes	No	No
**Tak et al. 2018**	RFA + Dox	RFA	NR	PFS	Yes	Yes	No	No
**Bruix et al. 2015**	Surgery + Sorafenib	Surgery	8.5	RFS	Yes	Yes	Yes	No

© 2024 Patel KR et al. *JAMA Network Open.*

**Lencioni et al. 2016**	TACE-DEB + Sorafenib	TACE-DEB	9.1	TTP	No	Yes	No	No
**Meyer et al. 2017**	TACE-DEB + Sorafenib	TACE-DEB	21	PFS	Yes	Yes	No	No
**Brown et al. 2016**	TAE	TACE-DEB	34	RTT	No	No	Yes	Yes
**Wang et al. 2018**	Surgery	Surgery + TACE	44.1	RFS	Yes	Yes	Yes	Yes
**Li et al. 2023**	Surgery	Surgery + FOLFOX HAI	23.7	DFS	Yes	Yes	Yes	Yes
**Sun et al. 2019**	Surgery	Surgery + EBRT (50 Gy/25 fx)	12	OS	Yes	Yes	Yes	Yes
**Li et al. 2020**	Surgery	Surgery + RAI	55.9	RFS	Yes	Yes	Yes	Yes
**Chen et al. 2013**	Surgery	Surgery + I-125 BT	47.6	TTP	Yes	Yes	Yes	Yes
**Rong et al. 2020**	Surgery	Surgery + EBRT (60 Gy/ 30 fx)	NR	RFS	Yes	Yes	Yes	Yes
**Shi et al. 2022**	Surgery	Surgery + SBRT (35 Gy/ 5 fx	52	DFS	Yes	Yes	Yes	Yes
**Wei et al. 2018**	Surgery	Surgery + TACE	37.5	DFS	Yes	Yes	Yes	Yes
**Vietti Violi et al. 2018**	MWA	RFA	26	LC	No	No	No	Yes

Abbreviations: RFA = radiofrequency ablation, EBRT = external beam radiation therapy, SBRT = stereotactic body radiation therapy, PBT = proton beam therapy, Gy= Gray (SI), fx = fraction, TACE = transarterial chemoembolization, TARE = transarterial radiofrequency embolization, 5FU = 5-fluorauracil, DEB = drug-eluting bead, FOLFOX = folinic acid, fluorouracil, and oxaliplatin, HAI = Hepatic arterial infusion, CDDP = cisplatin, LEN= lenvatinib, RFS = recurrence free survival, NR = not reported, LC = local control, RTT = Response to treatment, TTP = time to progression, LPFS = locoregional progression free survival, OS = overall survival, PFS = progression free survival,

© 2024 Patel KR et al. *JAMA Network Open.*

eFigure 1. Risk of Bias Assessment

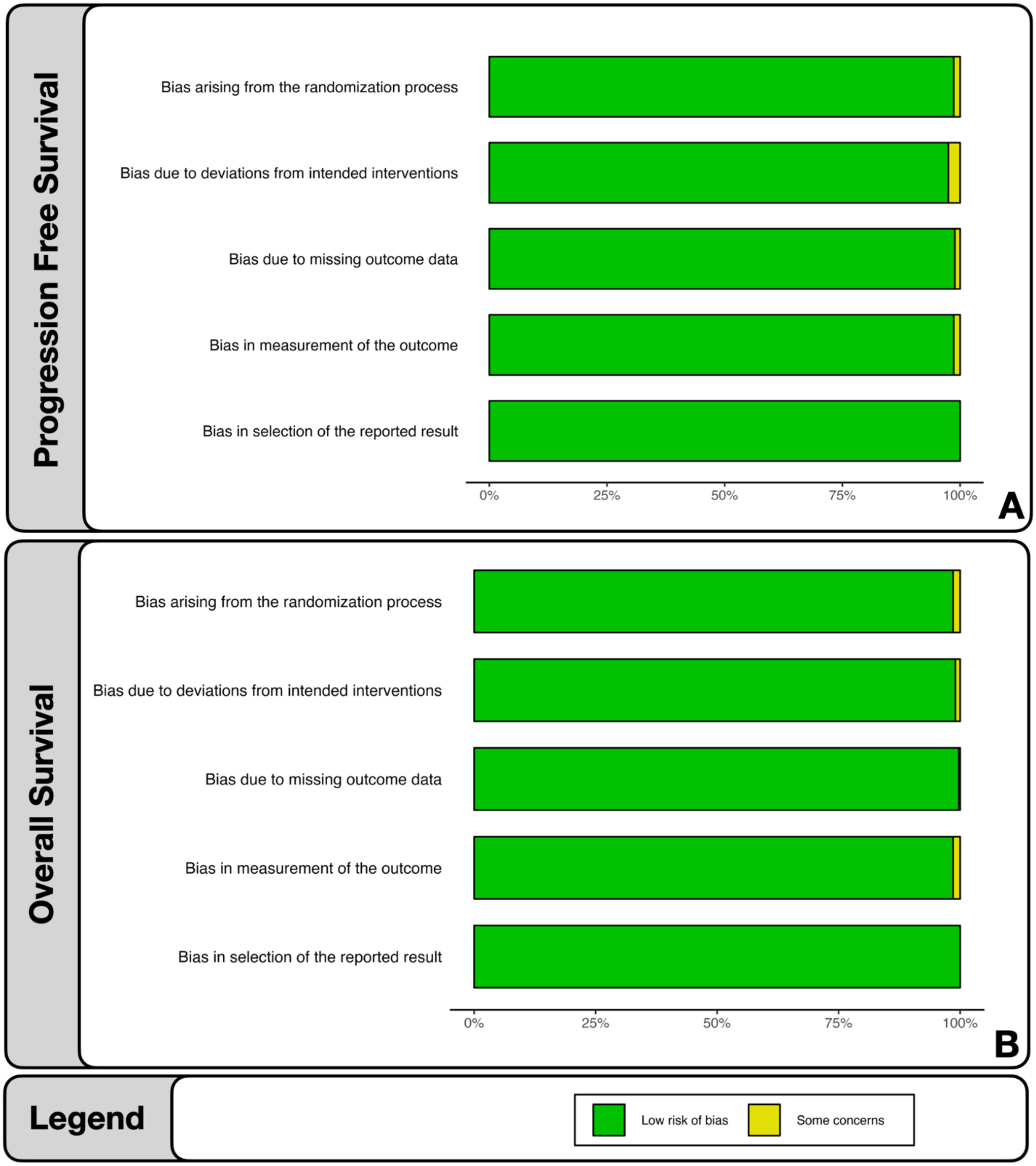

© 2024 Patel KR et al. *JAMA Network Open.*

eFigure 2. PRISMA Flowchart of Study Selection

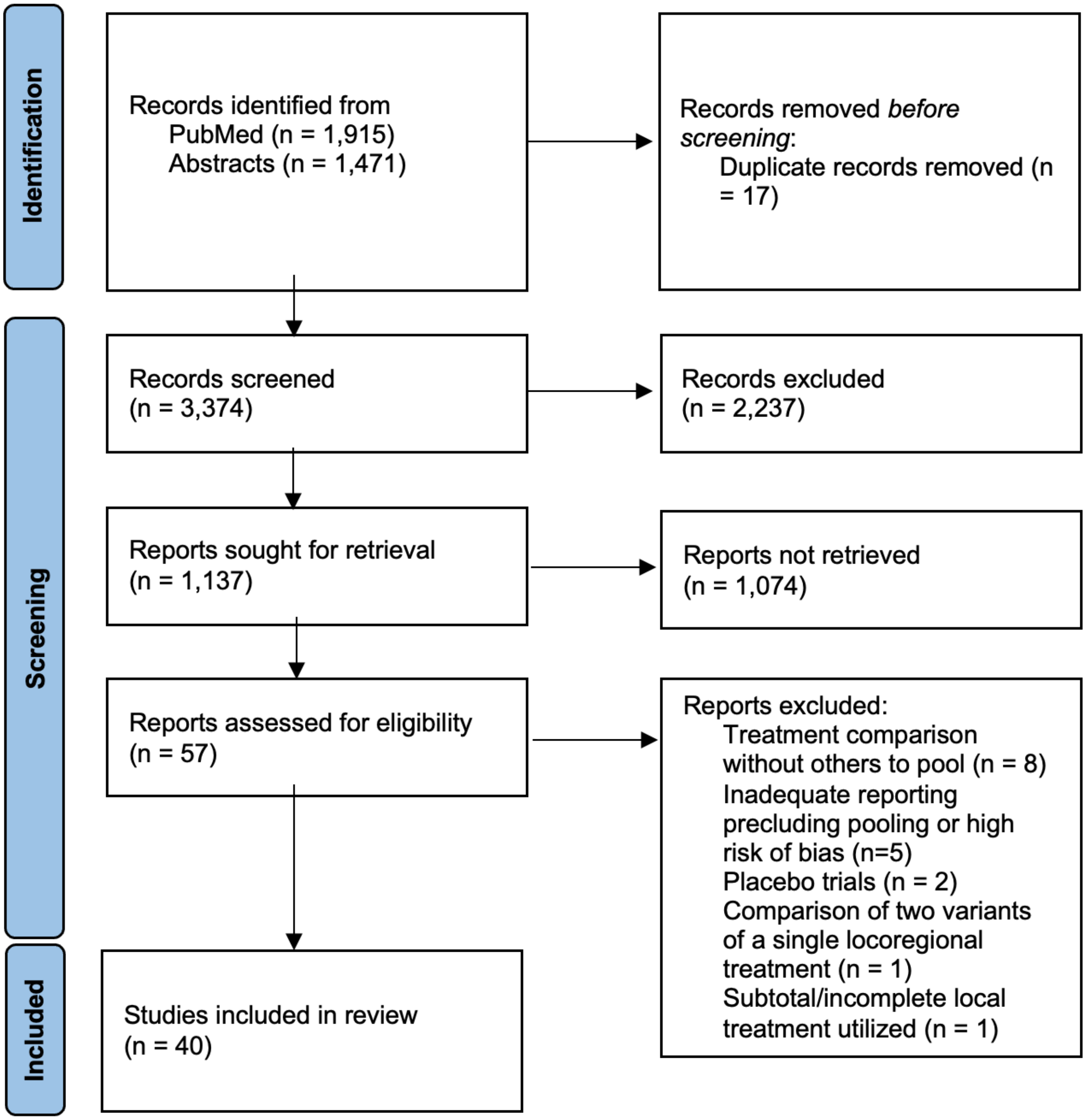

© 2024 Patel KR et al. *JAMA Network Open.*

eFigure 3. Surgery ± Adjuvant. Forest plots of studies comparing surgery with surgery plus additional LRT methods given in the adjuvant setting with corresponding meta-analytic estimates.

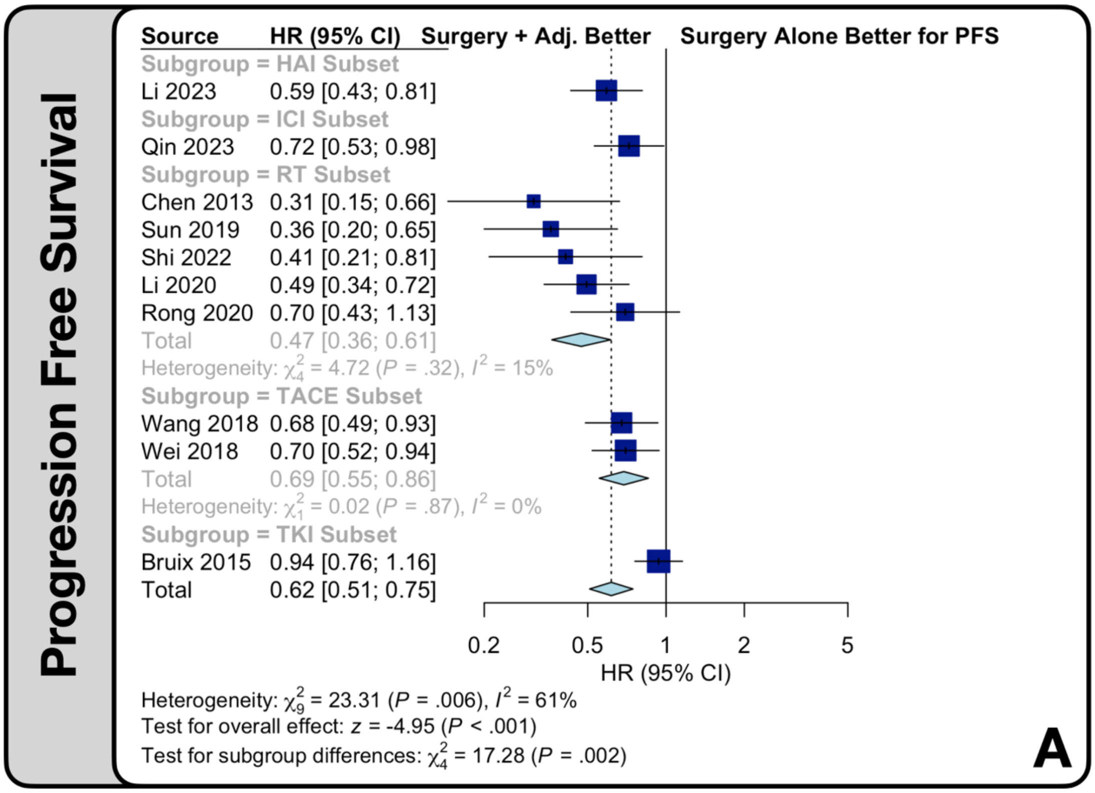

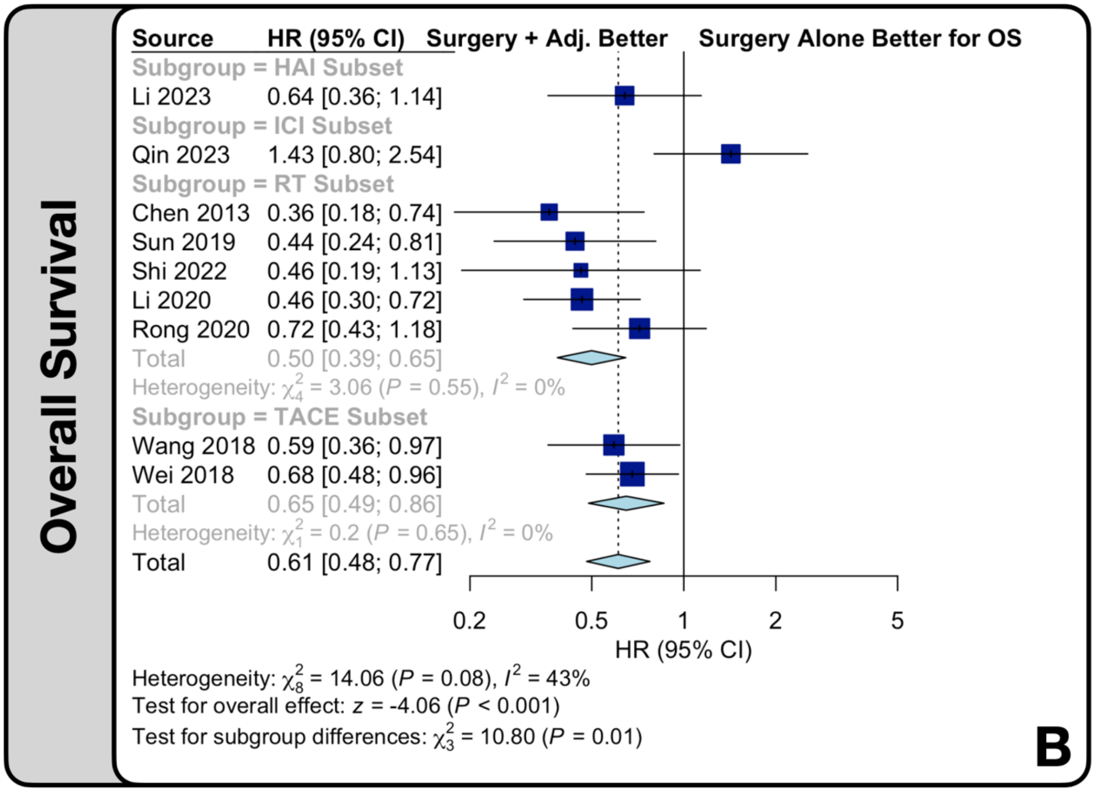

© 2024 Patel KR et al. *JAMA Network Open.*

eFigure 4. Surgery vs RFA. Forest plots of studies comparing surgery with radiofrequency ablation (RFA) with corresponding meta-analytic estimates.

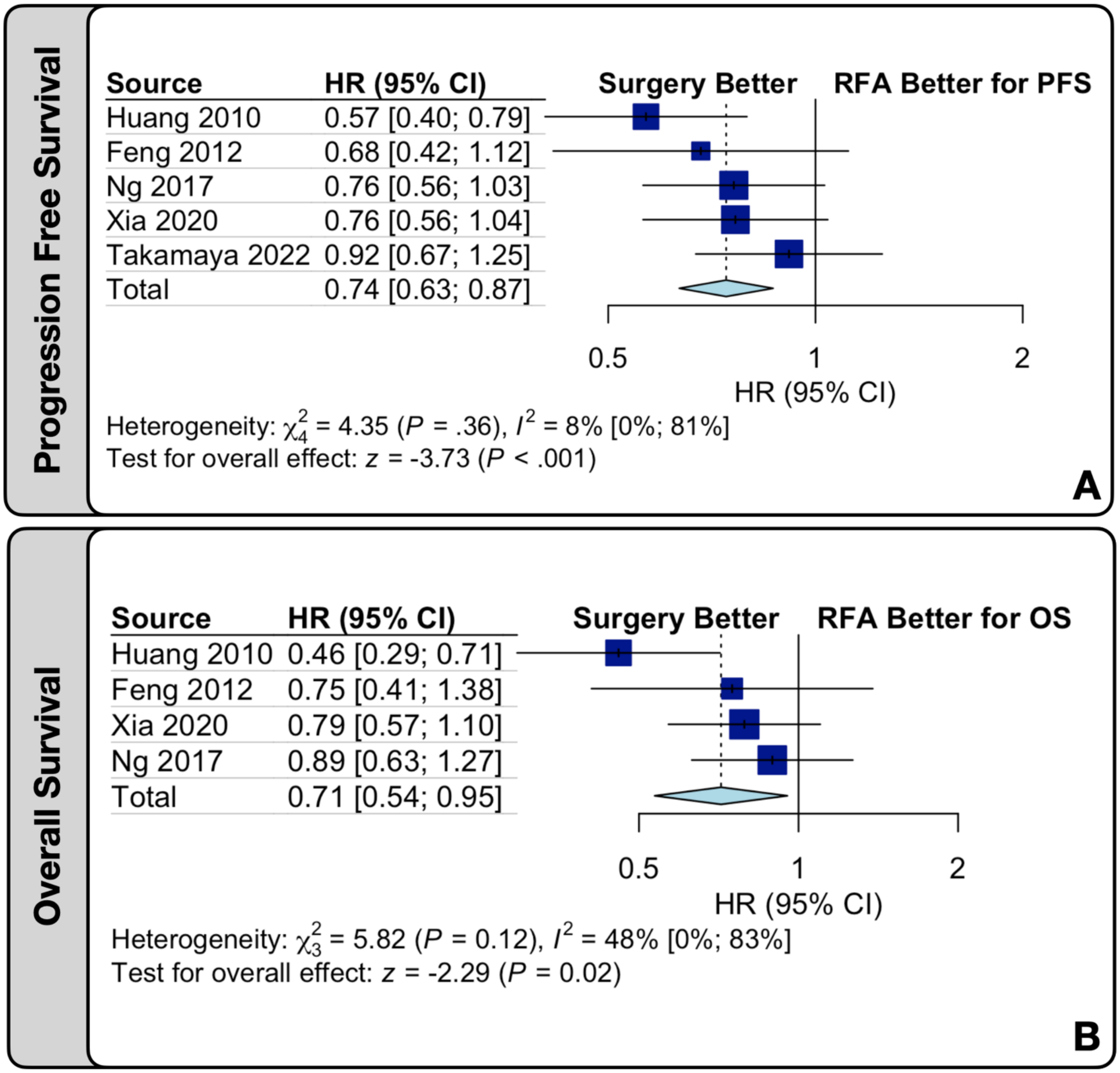

© 2024 Patel KR et al. *JAMA Network Open.*

eFigure 5. RT vs. Other. Forest plots of studies comparing radiotherapy (RT) with other locoregional therapies by subgroup with corresponding meta-analytic estimates.

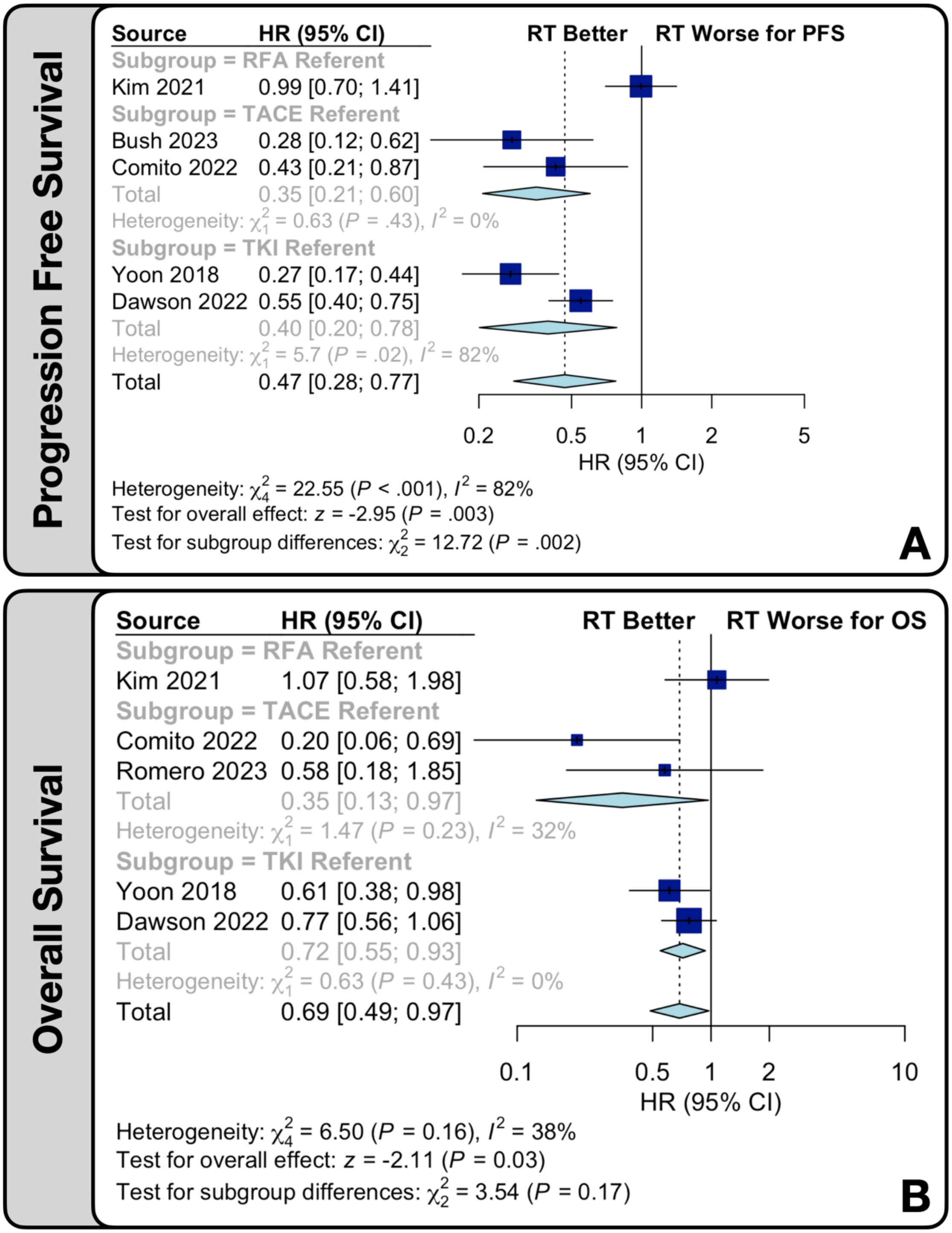

© 2024 Patel KR et al. *JAMA Network Open.*

eFigure 6. HAI vs Other. Forest plots of studies comparing hepatic arterial infusion (HAI) chemotherapy-based treatments with comparators by subgroup with corresponding meta-analytic estimates.

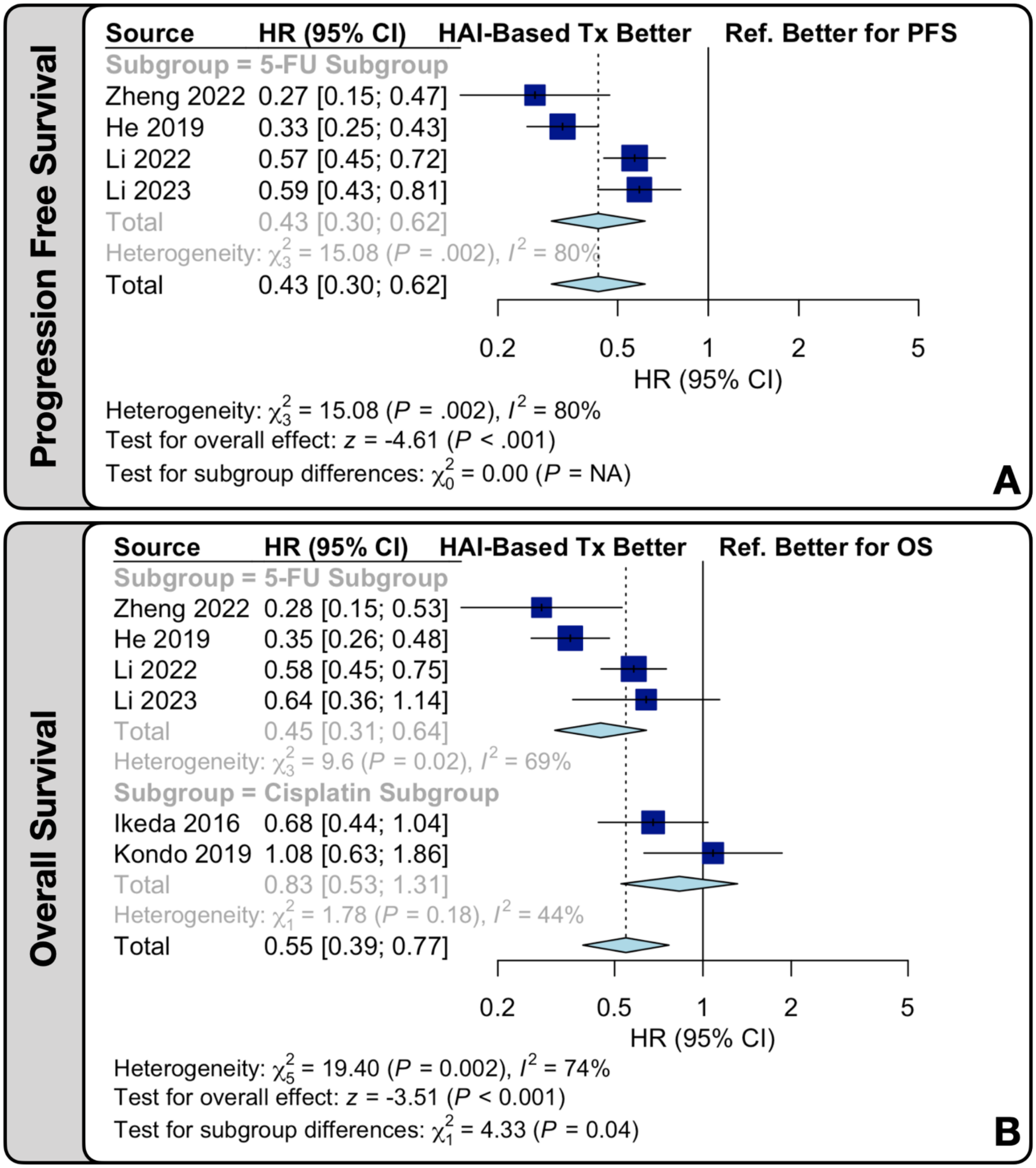

© 2024 Patel KR et al. *JAMA Network Open.*

eFigure 7. TACE vs. Other. Forest plots of studies comparing trans-arterial chemoembolization (TACE) comparators by subgroup with corresponding meta-analytic estimates.

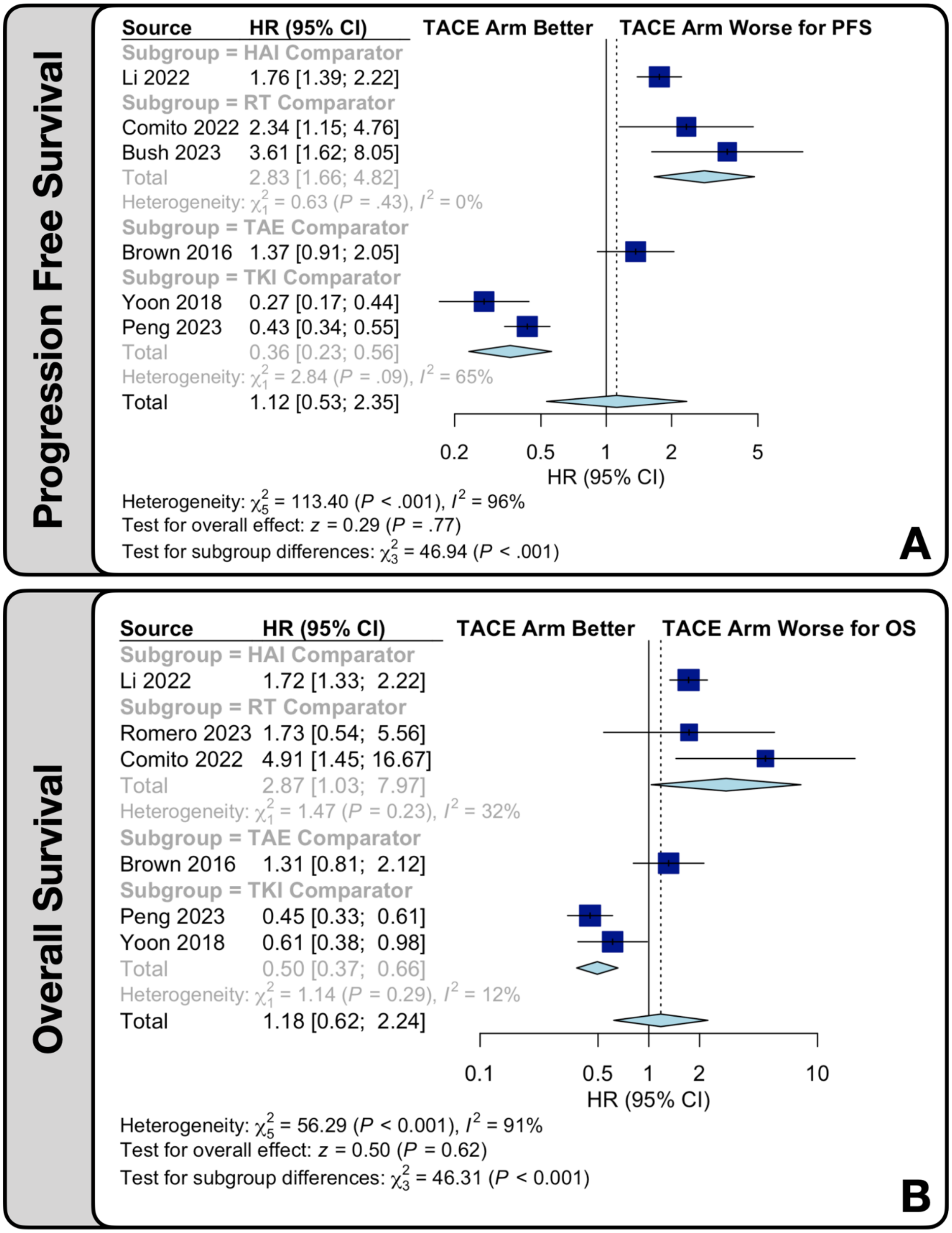

© 2024 Patel KR et al. *JAMA Network Open.*

eFigure 8. TARE vs TKI-Based Therapy. Forest plots of studies comparing trans-arterial radioembolization (TARE) comparators by subgroup with corresponding meta-analytic estimates.

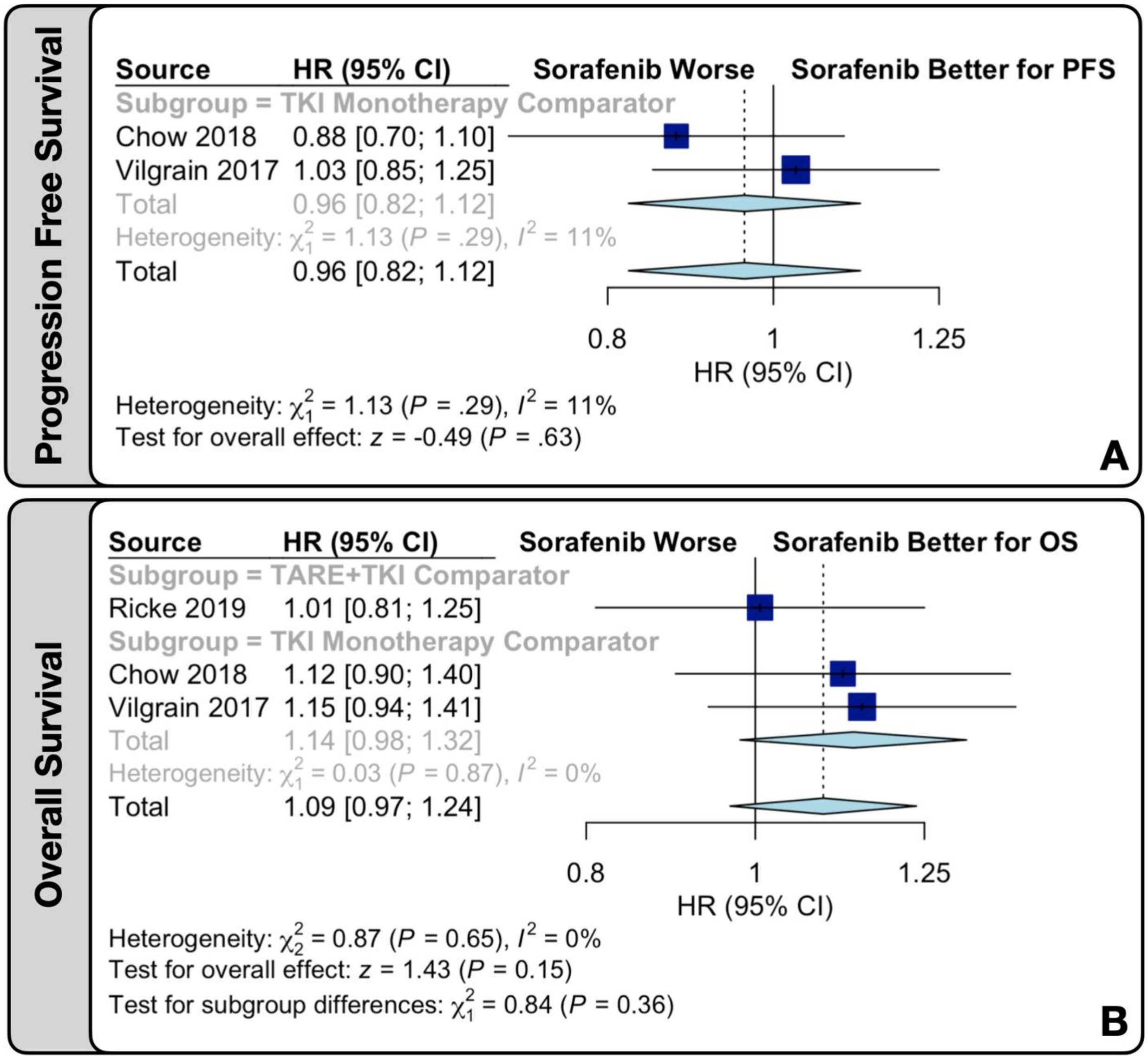

© 2024 Patel KR et al. *JAMA Network Open.*

eFigure 9. Network Meta-Analysis. Forest plot of a comparative efficacy estimates on the endpoints of PFS and OS against the referent of surgery from a network meta-analysis.

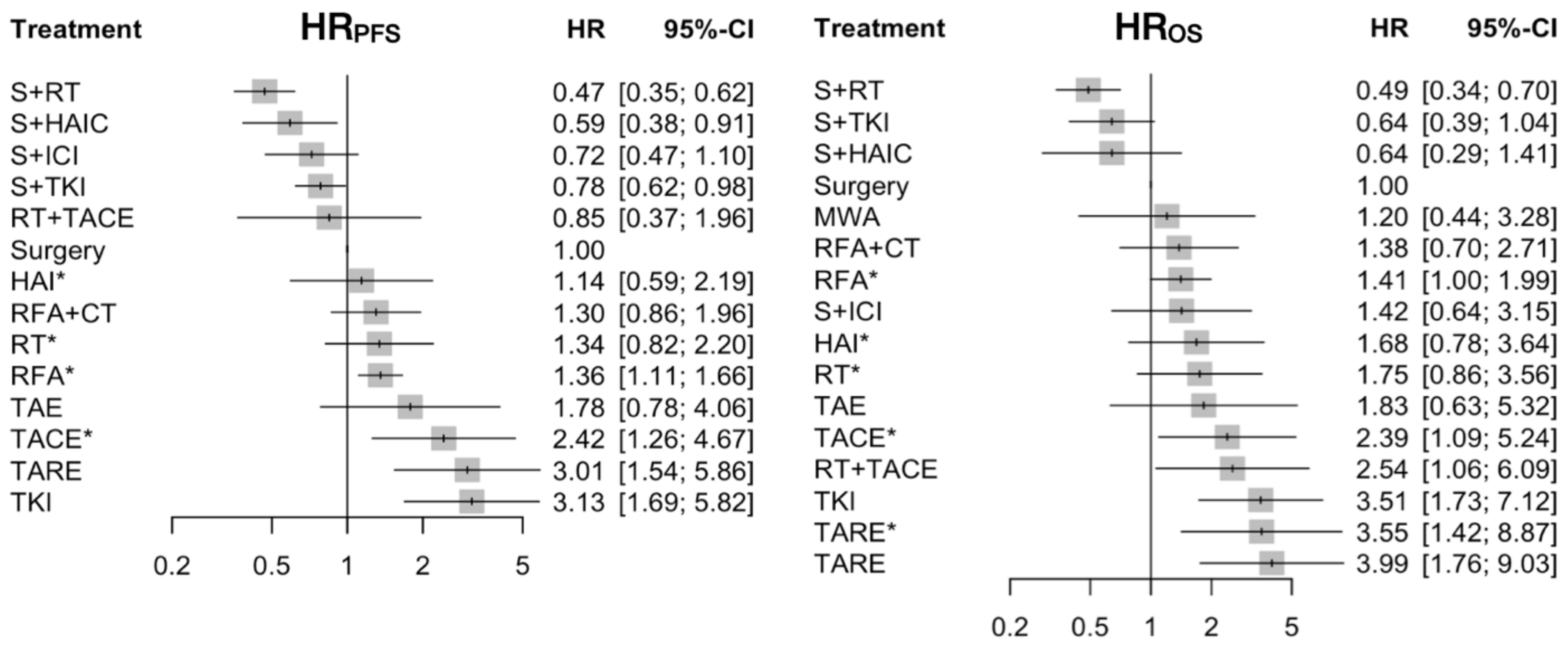

* Asterisk denotes locoregional therapy with or without additional treatments.

© 2024 Patel KR et al. *JAMA Network Open.*

eAppendix

RESULTS

The Comparative Role of Systemic and Locoregional Therapy (LRT)

Systemic Therapy ± LRT

Pooling studies that investigated locoregional intensification of systemic therapy with RFA, RT, HAIC, or embolization-based methods[4-11] showed that local intensification improved PFS (HR 0.40 [95% CI: 0.30-0.52]; p<0.001) and OS (HR 0.56 [95% CI: 0.40-0.81]; p=0.02) (**eAppendix Figure 1A**). Heterogeneity was observed in both analyses ($Q_{df=3}$=8.26, p=0.04 for PFS and $Q_{df=7}$=51.93, p<0.001 for OS). A subgroup analysis (**eAppendix Figure 2**) including a HAIC subgroup and another subgroup comprised of other forms of LRT was conducted, and the benefit of local intensification remained consistent across subgroups on both outcomes of PFS (both p<0.001) and OS (both p<0.05).

LRT ± Systemic Therapy

Pooling studies that investigated the addition of systemic therapy to LRT[12-19] showed an improvement in PFS (HR 0.90 [95% CI: 0.82-0.98], p=0.02), but no corresponding improvement in OS (HR 1.00 [95% CI: 0.90-1.11], p>0.99) (**eAppendix Figure 1B**). No significant heterogeneity was observed for PFS ($Q_{df=4}$=3.66, p=0.45) or OS ($Q_{df=7}$=3.24, p=0.86) between treatment class comparisons (**eAppendix Figure 3**).

LRT vs. Systemic Therapy

Pooling trials that compared local therapy alone to systemic therapy[20-22] did not detect a difference in PFS or OS between classes (**eAppendix Figure 1C**). As significant heterogeneity was observed ($Q_{df=2}$=25.72, p<0.001 for PFS and $Q_{df=2}$=6.07, p=0.048 for OS), subgroup analysis was conducted. The heterogeneity was a result of the inclusion of a single trial that showed a large benefit of intensive local treatment with TACE+RT over sorafenib[22]. In contrast, the other studies[20,21] comparing TARE to sorafenib showed no significant evidence of a difference between arms (**eAppendix Figure 4**).

A network meta-analysis was done showing a consistent ordinality of results (**eAppendix Figure 5**)

DISCUSSION

The Comparative Role of Systemic and Locoregional Therapy (LRT)

The comparative efficacy of systemic therapy alone versus LRT alone in practice observed may be a result of the baseline disease features between trials or differences in the efficacy of various LRTs, given the high heterogeneity observed. Intuitively, systemic therapy serves to improves extrahepatic control while LRT improves intrahepatic control, as observed in SARAH[21]. Thus, future work utilizing baseline imaging and patient factors to weigh the competing risk of intra- versus extra-hepatic progression may be important to determine which of these two should be considered a higher priority when clinicians are faced with such a decision in a patient eligible for both.

Based on the availability of the reported RCTs, we explored to see if the comparative efficacy of each treatment class (LRT, systemic therapy, or both) was congruent with known patterns of failure. Because many patients die of local disease burden and/or critical liver dysfunction rather than from metastatic disease, it was hypothesized that locoregional control is an important management priority for patients, and the differential efficacy of various LRTs was also discerned herein. First, we found evidence that the addition of LRT to systemic therapy likely improves both PFS and OS. This is consistent with the observation that the most significant pattern of failure in transplant-ineligible patients is local[23,24], and therefore, locoregional intensification may improve the disease-free interval for such patients. Given that the leading cause of death in patients with HCC is disease-related[25,26], this has the potential to translate into improved OS, as was observed herein. He et al.[6] showed that only 4% of patients demonstrated an intra-hepatic overall response rate (partial or complete) with sorafenib monotherapy, and, although novel immunotherapy agents have demonstrated an improved

© 2024 Patel KR et al. *JAMA Network Open.*

local activity of systemic therapy, the ORR continues to indicate suboptimal local control at approximately 15%[27]-20%[28] for immunotherapy alone and approximately 25-30% for immunotherapy in combination[29]. Based on the available evidence, the observed PFS benefit from the inclusion of LRT appears to translate to an improvement in OS, highlighting the importance of effective locoregional control in patients with non-metastatic HCC.

Additionally, our findings suggest that the addition of systemic therapy to LRT also improves PFS. This may occur via one of two mechanisms: (1) a decrease in distant metastases or (2) a decrease in intrahepatic foci outside the LRT volume, a failure pattern which may be more frequent than in-volume recurrence[30]. This increase in treatment intensity, however, did not translate into an improvement in OS. It is possible that because patients who fail LRT will almost always continue to be candidates for systemic therapy as disease progresses, there is little survival benefit to early systemic therapy. This contrasts with the prior case of upfront locoregional intensification of systemic therapy where patients may often lose eligibility to receive local ablation due to intrahepatic or extrahepatic progression, thus potentially missing the opportunity to experience the benefit of the addition of this form of treatment in their lifespan.

© 2024 Patel KR et al. *JAMA Network Open.*

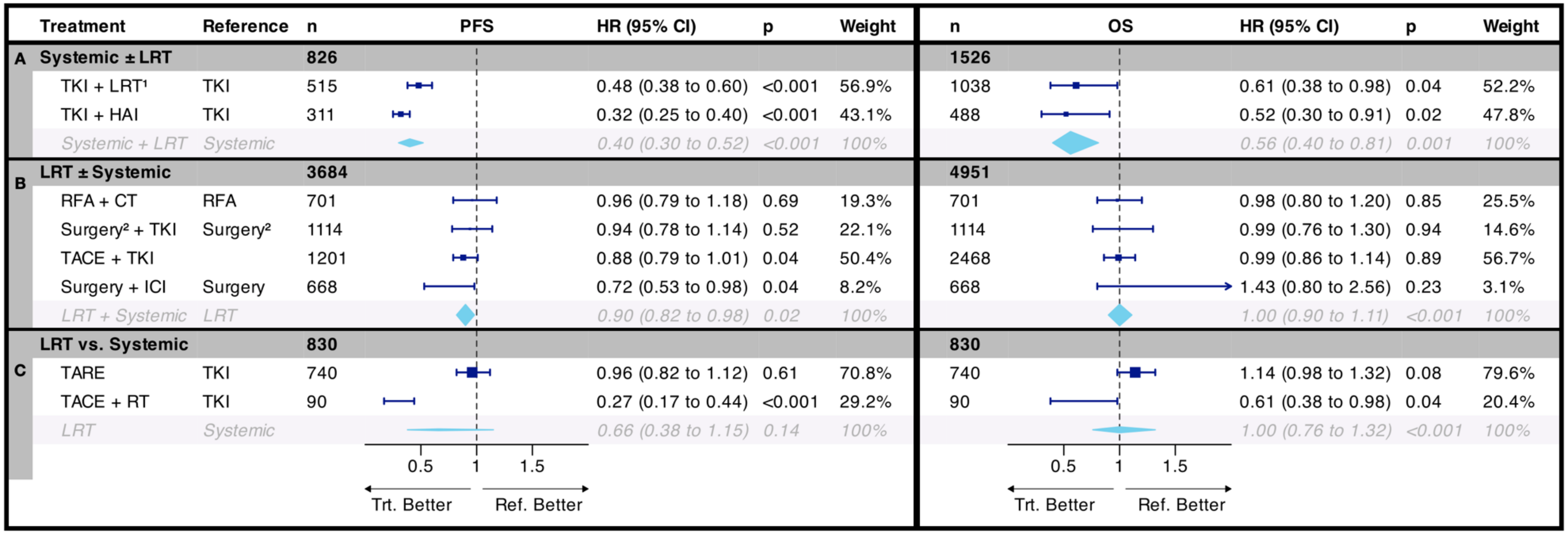

eAppendix Figure 1. Forest plots for the first analysis comparing classes of therapy.

[1] LRT consisting of RFA, SBRT, TACE, and TARE
[2] Estimate derived from the STORM trial[17] in which 900 of 1114 patients underwent surgery and the remaining 214 patients underwent other forms of loco-regional therapy. Given that the subgroup analysis revealed a very similar HR for PFS between the two groups and this study fulfilled the criteria for this analysis, these were pooled for this analysis. Of note, no subgroup analysis was reported for OS in this study.

Abbreviations: TKI: tyrosine kinase inhibitor; LRT: locoregional therapy; HAI: hepatic arterial infusion chemotherapy; RFA: Radiofrequency ablation; CT: Chemotherapy; TACE: Transarterial chemoembolization; TARE: Transarterial radioembolization; RT: radiotherapy; Trt.: Treatment; Ref.: Reference treatment; CI: confidence interval; PFS: progression-free survival; OS: overall survival

© 2024 Patel KR et al. *JAMA Network Open.*

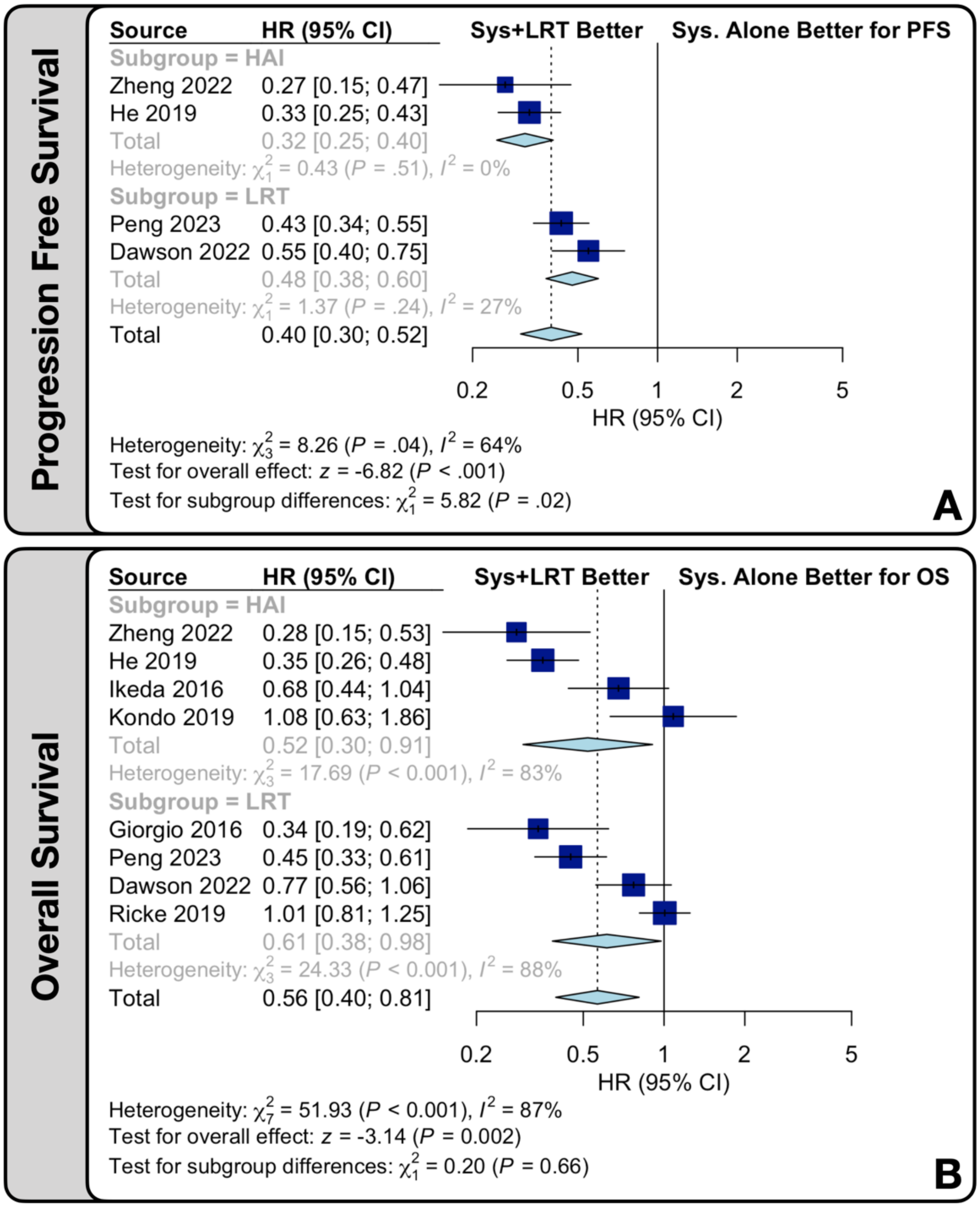

eAppendix Figure 2. Forest Plots Corresponding to the Meta-analysis of Systemic Therapy with or without Locoregional Therapy by Subgroup of Hepatic Arterial Infusion Chemotherapy Studies vs. Other Locoregional Therapy Studies.

© 2024 Patel KR et al. *JAMA Network Open.*

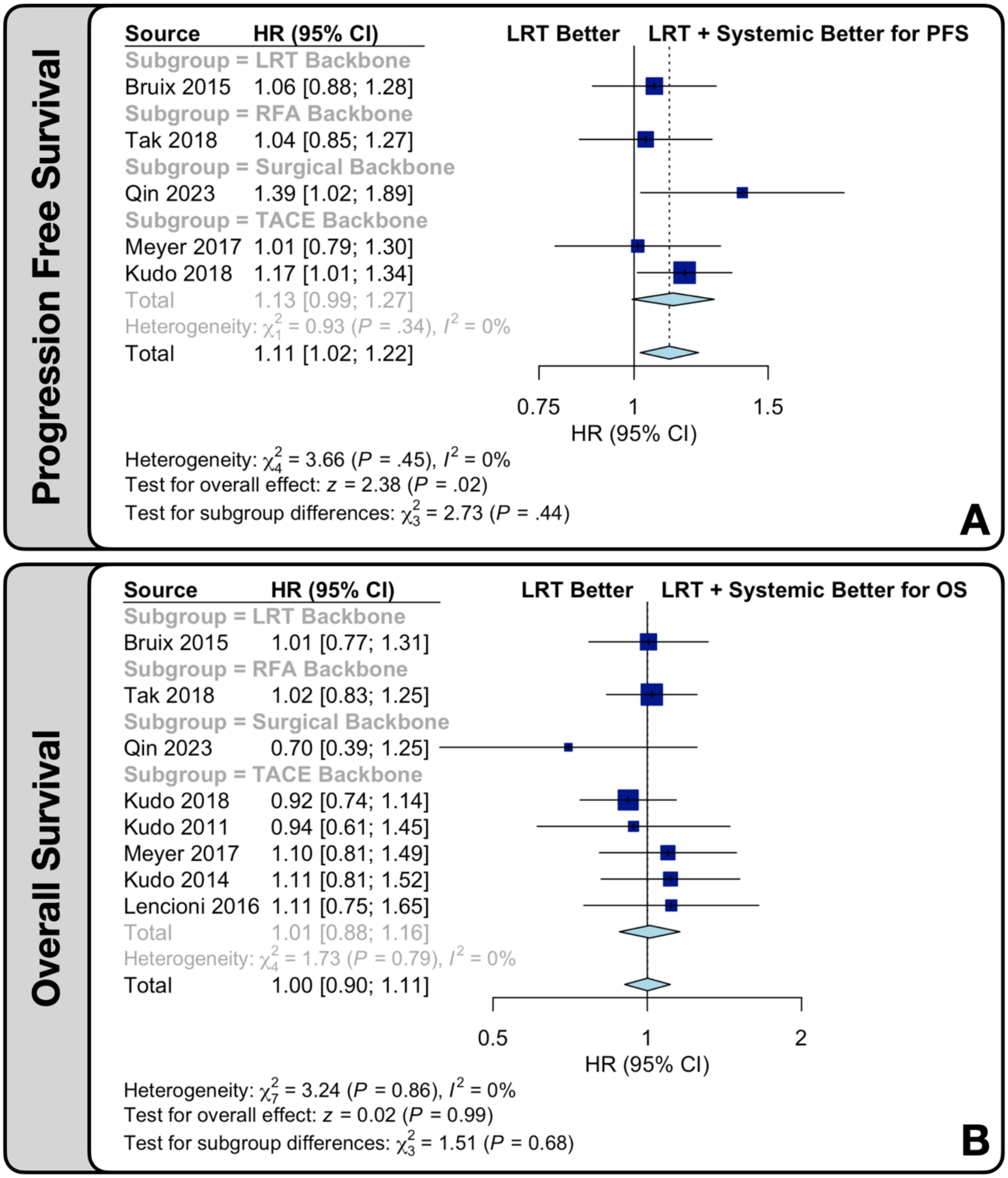

eAppendix Figure 3. Forest Plots Corresponding to the Meta-analysis of Locoregional Therapy with or without Systemic Therapy by Subgroup of Locoregional Therapy Backbone.

© 2024 Patel KR et al. *JAMA Network Open.*

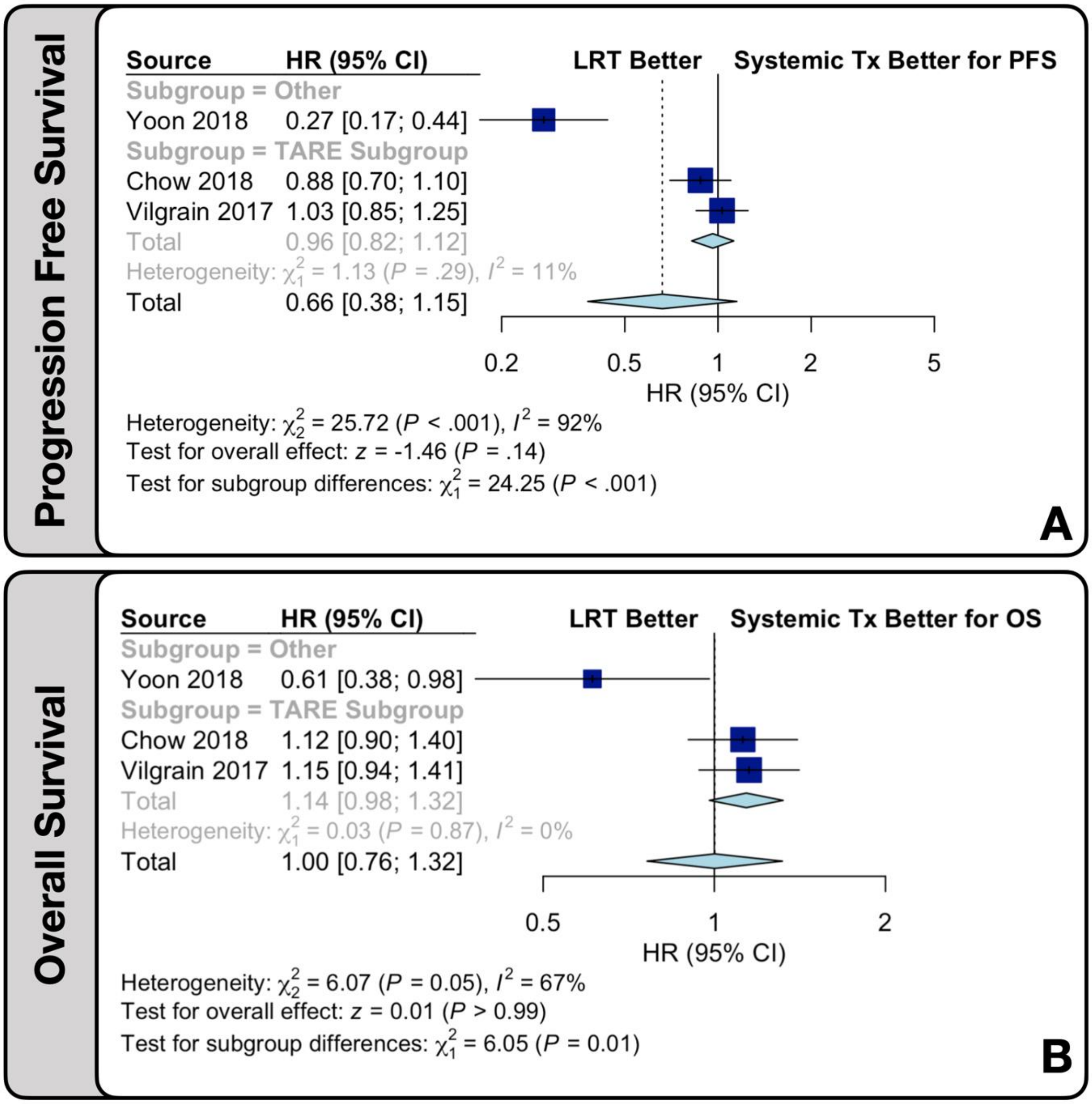

eAppendix Figure 4. Forest Plots Corresponding to the Meta-analysis of Locoregional Therapy Compared with Systemic Therapy Alone by Subgroup.

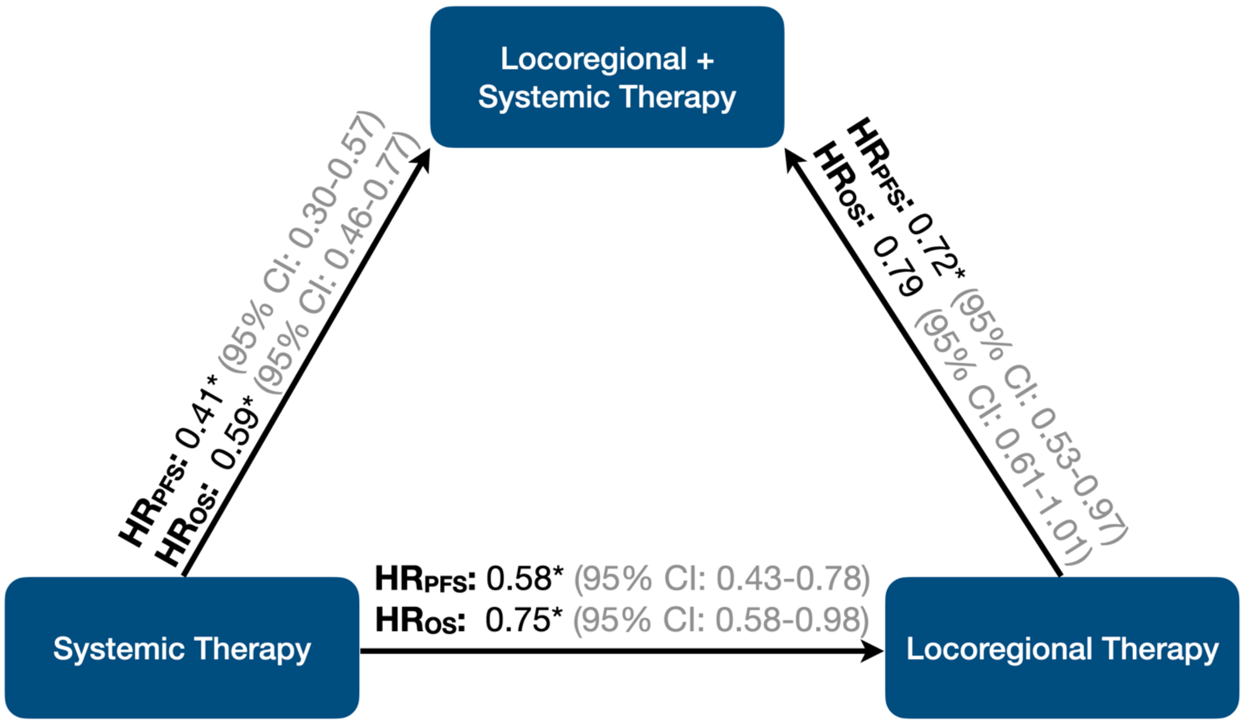

eAppendix Figure 5. Effect Estimates Determined from a full network meta-analysis utilizing both Direct and Indirect Evidence. Arrow heads indicate the favored treatment (i.e., the one with better overall or progression free survival) of the comparison. Effect estimates which were significant at the 0.05 threshold are denoted with an asterisk (*).

© 2024 Patel KR et al. *JAMA Network Open.*

eAppendix References

1. Network NCC. Hepatocellular Carcinoma (Version 2.2023). 2023. Accessed November 22, 2023, 2023. https://www.nccn.org/professionals/physician_gls/pdf/hcc.pdf
2. Kudo M, Kawamura Y, Hasegawa K, et al. Management of Hepatocellular Carcinoma in Japan: JSH Consensus Statements and Recommendations 2021 Update. *Liver cancer*. Jun 2021;10(3):181-223. doi:10.1159/000514174
3. Higgins JPT, Altman DG, Gøtzsche PC, et al. The Cochrane Collaboration's tool for assessing risk of bias in randomised trials. *BMJ*. 2011;343:d5928. doi:10.1136/bmj.d5928
4. Dawson LA, Winter K, Knox J, et al. NRG/RTOG 1112: Randomized Phase III Study of Sorafenib vs. Stereotactic Body Radiation Therapy (SBRT) Followed by Sorafenib in Hepatocellular Carcinoma (HCC) (NCT01730937). *International Journal of Radiation Oncology Biology Physics*. Dec 1 2022;114(5):1057-1057. doi:https://doi.org/10.1016/j.ijrobp.2022.09.002
5. Giorgio A, Merola MG, Montesarchio L, et al. Sorafenib Combined with Radio-frequency Ablation Compared with Sorafenib Alone in Treatment of Hepatocellular Carcinoma Invading Portal Vein: A Western Randomized Controlled Trial. *Anticancer Res*. Nov 2016;36(11):6179-6183. doi:10.21873/anticanres.11211
6. He M, Li Q, Zou R, et al. Sorafenib Plus Hepatic Arterial Infusion of Oxaliplatin, Fluorouracil, and Leucovorin vs Sorafenib Alone for Hepatocellular Carcinoma With Portal Vein Invasion: A Randomized Clinical Trial. *JAMA oncology*. Jul 1 2019;5(7):953-960. doi:10.1001/jamaoncol.2019.0250
7. Ikeda M, Shimizu S, Sato T, et al. Sorafenib plus hepatic arterial infusion chemotherapy with cisplatin versus sorafenib for advanced hepatocellular carcinoma: randomized phase II trial. *Ann Oncol*. Nov 2016;27(11):2090-2096. doi:10.1093/annonc/mdw323
8. Kondo M, Morimoto M, Kobayashi S, et al. Randomized, phase II trial of sequential hepatic arterial infusion chemotherapy and sorafenib versus sorafenib alone as initial therapy for advanced hepatocellular carcinoma: SCOOP-2 trial. *BMC cancer*. Oct 15 2019;19(1):954. doi:10.1186/s12885-019-6198-8
9. Peng Z, Fan W, Zhu B, et al. Lenvatinib Combined With Transarterial Chemoembolization as First-Line Treatment for Advanced Hepatocellular Carcinoma: A Phase III, Randomized Clinical Trial (LAUNCH). *Journal of clinical oncology : official journal of the American Society of Clinical Oncology*. Jan 1 2023;41(1):117-127. doi:10.1200/JCO.22.00392
10. Ricke J, Klumpen HJ, Amthauer H, et al. Impact of combined selective internal radiation therapy and sorafenib on survival in advanced hepatocellular carcinoma. *Journal of hepatology*. Dec 2019;71(6):1164-1174. doi:10.1016/j.jhep.2019.08.006
11. Zheng K, Zhu X, Fu S, et al. Sorafenib Plus Hepatic Arterial Infusion Chemotherapy versus Sorafenib for Hepatocellular Carcinoma with Major Portal Vein Tumor Thrombosis: A Randomized Trial. *Radiology*. May 2022;303(2):455-464. doi:10.1148/radiol.211545
12. Kudo M, Cheng A-L, Park J-W, et al. Orantinib versus placebo combined with transcatheter arterial chemoembolisation in patients with unresectable hepatocellular carcinoma (ORIENTAL): a randomised, double-blind, placebo-controlled, multicentre, phase 3 study. *The lancet Gastroenterology & hepatology*. 2018;3(1):37-46.
13. Kudo M, Han G, Finn RS, et al. Brivanib as adjuvant therapy to transarterial chemoembolization in patients with hepatocellular carcinoma: a randomized phase III trial. *Hepatology (Baltimore, Md)*. 2014;60(5):1697-1707.
14. Kudo M, Imanaka K, Chida N, et al. Phase III study of sorafenib after transarterial chemoembolisation in Japanese and Korean patients with unresectable hepatocellular carcinoma. *European journal of cancer*. 2011;47(14):2117-2127.
15. Meyer T, Fox R, Ma YT, et al. Sorafenib in combination with transarterial chemoembolisation in patients with unresectable hepatocellular carcinoma (TACE 2): a

© 2024 Patel KR et al. *JAMA Network Open.*

randomised placebo-controlled, double-blind, phase 3 trial. *The lancet Gastroenterology & hepatology*. Aug 2017;2(8):565-575. doi:10.1016/s2468-1253(17)30156-5
16. Qin S, Chen M, Cheng A-L, et al. Atezolizumab plus bevacizumab versus active surveillance in patients with resected or ablated high-risk hepatocellular carcinoma (IMbrave050): a randomised, open-label, multicentre, phase 3 trial. *The Lancet*. 2023;402(10415):1835-1847.
17. Bruix J, Takayama T, Mazzaferro V, et al. Adjuvant sorafenib for hepatocellular carcinoma after resection or ablation (STORM): a phase 3, randomised, double-blind, placebo-controlled trial. *The lancet oncology*. 2015;16(13):1344-1354.
18. Tak WY, Lin SM, Wang Y, et al. Phase III HEAT Study Adding Lyso-Thermosensitive Liposomal Doxorubicin to Radiofrequency Ablation in Patients with Unresectable Hepatocellular Carcinoma Lesions. *Clinical cancer research : an official journal of the American Association for Cancer Research*. Jan 1 2018;24(1):73-83. doi:10.1158/1078-0432.Ccr-16-2433
19. Lencioni R, Llovet JM, Han G, et al. Sorafenib or placebo plus TACE with doxorubicin-eluting beads for intermediate stage HCC: The SPACE trial. *Journal of hepatology*. May 2016;64(5):1090-1098. doi:10.1016/j.jhep.2016.01.012
20. Chow PKH, Gandhi M, Tan SB, et al. SIRveNIB: Selective Internal Radiation Therapy Versus Sorafenib in Asia-Pacific Patients With Hepatocellular Carcinoma. *Journal of clinical oncology : official journal of the American Society of Clinical Oncology*. Jul 1 2018;36(19):1913-1921. doi:10.1200/JCO.2017.76.0892
21. Vilgrain V, Pereira H, Assenat E, et al. Efficacy and safety of selective internal radiotherapy with yttrium-90 resin microspheres compared with sorafenib in locally advanced and inoperable hepatocellular carcinoma (SARAH): an open-label randomised controlled phase 3 trial. *The Lancet Oncology*. Dec 2017;18(12):1624-1636. doi:10.1016/S1470-2045(17)30683-6
22. Yoon SM, Ryoo BY, Lee SJ, et al. Efficacy and Safety of Transarterial Chemoembolization Plus External Beam Radiotherapy vs Sorafenib in Hepatocellular Carcinoma With Macroscopic Vascular Invasion: A Randomized Clinical Trial. *JAMA oncology*. May 1 2018;4(5):661-669. doi:10.1001/jamaoncol.2017.5847
23. Abdelaziz A, Elbaz T, Shousha HI, et al. Efficacy and survival analysis of percutaneous radiofrequency versus microwave ablation for hepatocellular carcinoma: an Egyptian multidisciplinary clinic experience. *Surg Endosc*. Dec 2014;28(12):3429-34. doi:10.1007/s00464-014-3617-4
24. Wang Z, Ren Z, Chen Y, et al. Adjuvant Transarterial Chemoembolization for HBV-Related Hepatocellular Carcinoma After Resection: A Randomized Controlled Study. *Clinical Cancer Research*. 2018;24(9):2074-2081. doi:10.1158/1078-0432.Ccr-17-2899
25. Llovet JM, Kelley RK, Villanueva A, et al. Hepatocellular carcinoma. *Nature reviews Disease primers*. Jan 21 2021;7(1):6. doi:10.1038/s41572-020-00240-3
26. Yen YH, Kee KM, Li WF, et al. Causes of Death among Patients with Hepatocellular Carcinoma According to Chronic Liver Disease Etiology. *Cancers*. Mar 9 2023;15(6)doi:10.3390/cancers15061687
27. Qin S, Kudo M, Meyer T, et al. Tislelizumab vs Sorafenib as First-Line Treatment for Unresectable Hepatocellular Carcinoma: A Phase 3 Randomized Clinical Trial. *JAMA oncology*. 2023;doi:10.1001/jamaoncol.2023.4003
28. Abou-Alfa GK, Lau G, Kudo M, et al. Tremelimumab plus durvalumab in unresectable hepatocellular carcinoma. *NEJM evidence*. 2022;1(8):EVIDoa2100070.
29. Finn RS, Qin S, Ikeda M, et al. Atezolizumab plus Bevacizumab in Unresectable Hepatocellular Carcinoma. *The New England journal of medicine*. May 14 2020;382(20):1894-1905. doi:10.1056/NEJMoa1915745
30. Yoo GS, Yu JI, Cho S, et al. Comparison of clinical outcomes between passive scattering versus pencil-beam scanning proton beam therapy for hepatocellular carcinoma.

© 2024 Patel KR et al. *JAMA Network Open.*

Radiotherapy and oncology : journal of the European Society for Therapeutic Radiology and Oncology. May 2020;146:187-193. doi:10.1016/j.radonc.2020.02.019

© 2024 Patel KR et al. *JAMA Network Open.*
