# Supplementary material for: Locoregional Therapies for Hepatocellular Carcinoma: A Systematic Review and Meta-Analysis
Source: JAMA Netw Open. 2024 Nov 27;7(11):e2447995. doi: 10.1001/jamanetworkopen.2024.47995 (PMC12527482; doi:10.1001/jamanetworkopen.2024.47995)
Supplement: Supplement 2. — Data Sharing Statement [file jamanetwopen-e2447995-s002.pdf]

Data Sharing Statement

Patel. Locoregional Therapies for Hepatocellular Carcinoma. *JAMA Netw Open*. Published November 27, 2024. doi:10.1001/jamanetworkopen.2024.47995

Data
Data available: No

Additional Information
Explanation for why data not available: All data utilized in this study is publicly available. Data may be made available upon reasonable request to the corresponding author.
